# Antiangiogenic antibody BD0801 combined with immune checkpoint inhibitors achieves synergistic antitumor activity and affects the tumor microenvironment

**DOI:** 10.1186/s12885-021-08859-5

**Published:** 2021-10-22

**Authors:** Liting Xue, Xingyuan Gao, Haoyu Zhang, Jianxing Tang, Qian Wang, Feng Li, Xinxin Li, Xiaohong Yu, Zhihong Lu, Yue Huang, Renhong Tang, Wenqing Yang

**Affiliations:** 1 State Key Laboratory of Translational Medicine and Innovative Drug Development, Jiangsu Simcere Pharmaceutical Co. Ltd, Nanjing, Jiangsu China; 2 DMPK and Clinical Pharmacology, Suzhou Ribo Life Science Co. Ltd, Kushan, Jiangsu China; 3 Green Valley Research Institute, Shanghai Green Valley Pharmaceutical Co., Ltd, Shanghai, China

**Keywords:** Anti-VEGF monoclonal antibody, Immune checkpoint blockade, Combination treatment, Tumor microenvironment, Antitumor synergy

## Abstract

**Background:**

Signaling through VEGF/VEGFR induces cancer angiogenesis and affects immune cells. An increasing number of studies have recently focused on combining anti-VEGF/VEGFR agents and immune checkpoint inhibitors (ICIs) to treat cancer in preclinical and clinical settings. BD0801 is a humanized rabbit anti-VEGF monoclonal antibody in the clinical development stage.

**Methods:**

In this study, the anti-cancer activities of BD0801 and its potential synergistic anti-tumor effects when combined with different immunotherapies were assessed by using in vitro assays and in vivo tumor models. Ex vivo studies were conducted to reveal the possible mechanisms of actions (MOA) underlying the tumor microenvironment modification.

**Results:**

BD0801 showed more potent antitumor activity than bevacizumab, reflected by stronger blockade of VEGF/VEGFR binding and enhanced inhibitory effects on human umbilical vein endothelial cells (HUVECs). BD0801 exhibited dose-dependent tumor growth inhibitory activities in xenograft and murine syngeneic tumor models. Notably, combining BD0801 with either anti-PD-1 or anti-PD-L1 antibodies showed synergistic antitumor efficacy in both lung and colorectal cancer mouse models. Furthermore, the mechanistic studies suggested that the MOA of the antitumor synergy involves improved tumor vasculature normalization and enhanced T-cell mediated immunity, including increased tumor infiltration of CD8^+^ and CD4^+^ T cells and reduced double-positive CD8^+^PD-1^+^ T cells.

**Conclusions:**

These data provide a solid rationale for combining antiangiogenic agents with immunotherapy for cancer treatment and support further clinical development of BD0801 in combination with ICIs.

**Supplementary Information:**

The online version contains supplementary material available at 10.1186/s12885-021-08859-5.

## Background

Angiogenesis is a tightly regulated process and plays an important role in physiology and various pathologies [1, 2]. The concept of blocking angiogenesis for cancer therapy is well accepted in translational research and clinical development [3, 4]. Among tumor-derived angiogenic factors and cytokines, vascular endothelial growth factor A (VEGF-A, also called VEGF) is the major mediator of tumor angiogenesis, specifically the two circulating isoforms-VEGF_121_ and VEGF_165_ signaling through VEGF receptor 2 (VEGFR-2) [1, 4–6]. Both tumor cells and surrounding stromal cells secrete VEGF to stimulate the proliferation and survival of endothelial cells and form new blood vessels [7]. VEGF is expressed in most human cancers, and elevated VEGF expression levels are often related to a less favorable prognosis in cancer patients [6, 8]. Bevacizumab is the first US Food & Drug Administration (FDA)-approved recombinant humanized anti-VEGF monoclonal antibody for the treatment of non-small cell lung cancer (NSCLC), metastatic breast cancer, metastatic colorectal cancer, and other solid tumors [9].

Due to the complexity of cancer biology and interactions between the cancer cells and their microenvironment, effective anti-cancer therapies use combinatorial approaches to achieve greater efficacy in cancer patients instead of relying on a single agent or signaling pathway. Notably, besides sustained angiogenesis, immunosuppression is one of the hallmarks of cancer development and progression [10]. Cancer cells develop several escape mechanisms to evade the immune system, including induction of regulatory T (Treg) cells and myeloid-derived suppressor cells (MDSCs) and promotion of T cell exhaustion [11]. The inhibition of immune checkpoint regulators using antibodies targeting the cytotoxic T lymphocyte antigen 4 (CTLA-4), programmed cell death protein 1 (PD-1), and its ligand, programmed cell death ligand 1 (PD-L1), can stimulate the immune system and can induce sustained antitumor responses [12, 13]. The FDA approved ipilimumab (an anti-CTLA antibody) to treat melanoma patients in 2011 [14]. Nowadays, for example, nivolumab (an anti-PD-1 antibody) has been approved by the FDA to treat colorectal cancer, hepatocellular carcinoma (HCC), melanoma, lung cancer, and several other cancers [15, 16]. Besides, the FDA approved atezolizumab (an anti-PD-L1 antibody) to treat urothelial carcinoma, triple-negative breast cancer, and lung cancer [17, 18]. Still, the objective response rates (ORRs) to immune checkpoint inhibitors (ICIs) are not high, ranging from 10 to 40% in most solid tumors [19]. Therefore, numerous studies have been focusing on the mechanisms of resistance and the combination strategies for ICIs [12, 20, 21].

The interplay between immune suppression and upregulation of angiogenic pathways has been documented in the literature. Recent studies have shown that VEGF/VEGFR signaling can affect immune cells [22, 23]. VEGF increases the proliferation and homing of Treg cells, suppresses the maturation of dendritic cells and induces the expression of PD-L1 on dendritic cells. Besides, VEGF can suppress the proliferation of CD8^+^ T cells and enhance the expression of PD-1 and/or other inhibitory checkpoints such as T-cell immunoglobulin mucin 3 (TIM-3), lymphocyte activation gene-3 (LAG-3), and CTLA-4, leading to CD8^+^ T cell exhaustion [24]. On the other hand, nearly all the tumor-associated immune cells can support tumor angiogenesis [25]. Blockade of VEGF/VEGFR signaling can improve anti-PD-1 or anti-PD-L1 antibodies’ antitumor activities in murine tumor models of colorectal, pancreatic, breast, and small cell lung cancer [24, 26–28]. Furthermore, results of recent clinical investigations also support the enhanced antitumor activities by a combination of atezolizumab and bevacizumab in NSCLC [29], advanced renal cancer (ClinicalTrials.gov #NCT01984242/IMmotion150), and HCC patients (ClinicalTrials.gov #NCT02715531).

BD0801 is a humanized rabbit anti-VEGF monoclonal antibody developed by Simcere Pharma (Nanjing, China) and is now in phase 3 clinical studies (PCT patent application #PCT/CN 201080018409.6, NCT04908787). BD0801 can bind to human VEGF with a similar binding affinity as bevacizumab, although the two antibodies have different epitopes (unpublished data). BD0801 can inhibit HCC cell growth in vitro and induce cancer cell apoptosis more efficiently than bevacizumab [30]. In addition, the combination of BD0801 and chemotherapy was able to achieve better tumor growth inhibition than single-agent treatment in colorectal cancer mouse models [31]. Still, the combination of BD0801 and immunotherapy has yet to be explored.

The study investigated the feasibility and effectiveness of combining BD0801 with ICIs using lung cancer and colorectal cancer mouse models to support further clinical development of BD0801. Furthermore, mechanistic studies were conducted to reveal the mechanism of action (MOA) of the synergy.

## Methods

### Cell lines

For the HUVEC proliferation, apoptosis, and cell cycle analysis assays, HUVECs (Sciencell Research Laboratories, Carlsbad, CA, USA, #8000) were cultured in ECM medium (ScienCell, #1001) containing 5% FBS (ScienCell, #0025), 1% penicillin/streptomycin (GIBCO, Invitrogen Inc., Carlsbad, CA, USA, #15140–122) and 1% ECGS (ScienCell, #1052). For HUVEC Western blot, migration inhibition, and scratch assays, HUVECs (AllCells, Alameda, CA, USA) were maintained in HUVEC medium (Allcells, #H004B) with 10% FBS (Allcells, #H005) and HUVEC growth factors (Allcells, #H005). HUVECs at passages 4–8 were used in the experiments. The human non-small cell lung cancer cell line PC9 was purchased from Riken BioResource Research Center (Ibaraki, Japan, #RCB4455). The mouse lung cancer cell line 3LL was purchased from JCRB Cell Bank of the National Institutes of Biomedical Innovation, Health, and Nutrition (Tokyo, Japan, #JCRB-1348). The mouse colorectal cell line CT26 was purchased from the American Type Culture Collection (ATCC) (Manassas, VA, USA, #CRL-2638). The PC9, 3LL, and CT26 cells were cultured in RPMI-1640 medium (Gibco, #22400–089) with 10% FBS (Hyclone, Thermo Fisher Scientific, Waltham, MA, USA, #SV30087.03), 100 U/ml penicillin and 100 μg/ml streptomycin (Gibco, #15240–062).

### ELISA-based assays

#### VEGF/VEGFR2 binding blocking assay

Blank ELISA plates were coated with VEGFR2-his (Sino Biuological Inc., Beijing, China, #HPLC-10012-H08H) and blocked with 1% BSA in PBS. Different concentrations of BD0801 (Jiangsu Simcere Pharmaceutical Co., Ltd., Nanjing, China) or bevacizumab (Genentech/Roche, San Francisco, CA, USA) were incubated with human VEGF_165_ (PrimeGene, Shanghai, China, #105–05) at 37 °C for 1 h before adding into the plates coated with VEGFR2-his for incubation. Unbounded VEGF_165_ was washed off, and the primary antibody anti-VEGF rabbit mAb (Sino Biological, #11066-R105) was added for incubation. After washing the plates, the secondary antibody Peroxidase AffiniPure Donkey Anti-Rabbit IgG Jackson (Jackson ImmunoResearch, West Grove, PA, USA, #711–035-152) was added. After incubation, the redundant HRP complex was washed off. Finally, peroxidase (HRP) substrate TMB (Thermo Fisher Scientific, Waltham, MA, USA, #34029) was added, and the OD value was measured by SpectraMax I3X (Molecular Devices, LLC, Sunnyvale, CA, USA). The detection wavelength was 450 nm, and the reference wavelength was 630 nm. The IC_50_ was calculated based on the OD value at different concentrations by GraphPad Prism 8.0 (GraphPad Software Inc., San Diego, CA, USA). Each concentration was measured in duplicates in each experiment, and the experiment was repeated three times.

#### ELISA binding assay

Blank ELISA plates were coated with human VEGF_165_ (PrimeGene, Shanghai, China, #105–05), rat VEGF_164_ (PrimeGene, #145–07), or mouse VEGF_164_ (PrimeGene, #125–07) and blocked with 1% BSA in PBS. The different BD0801 or bevacizumab concentrations were added to the plates and unbound BD0801 or bevacizumab was washed off. The peroxidase affiniPure donkey anti-human IgG (Jackson ImmunoResearch, West Grove, PA, USA, #709–035-149) was added. After incubation, the redundant HRP complex was washed off. Finally, HRP substrate TMB (Thermo Fisher Scientific, Waltham, MA, USA, #34029) was added, and the OD value was measured by SpectraMax I3X. The detection wavelength was 450 nm, and the reference wavelength was 630 nm. The EC_50_ was calculated based on the OD value at different concentrations by GraphPad Prism 8.0. Each concentration was measured in triplicates.

#### ELISA analysis for BD0801 concentration in mouse serum

Blank ELISA plates were coated with VEGF_165_ (Sino Biuological Inc., Beijing, China, #11066-HNAB), and the serum samples were added. BD0801 in the serum samples were bound to VEGF, and the unbound BD0801 was washed off. Peroxidase AffiniPure donkey anti-human IgG (Jackson ImmunoResearch, West Grove, PA, USA, #709–035-149) was added. After incubation, redundant HRP complexes were washed off. HRP substrate TMB (R&D Systems, Minneapolis, MN, USA, #BMS258/2) was added. The OD value was measured using a microplate reader (TECAN, Männedorf, Switzerland). The detection wavelength was 450 nm, and the reference wavelength was 620 nm. Each sample was measured in duplicates. The concentration calculation of BD0801 for standard curve, quality control, and serum samples was performed by Microsoft Excel 2010 and SigmaPlot10.0. The standard curve fitting equation:

$$ \mathrm{y}=\mathrm{D}+\left(\mathrm{A}\hbox{-} \mathrm{D}\right)/\left(1+10\hat{\mkern6mu} \left(\left(\mathrm{x}\hbox{-} \mathrm{logC}\right)\ast \mathrm{B}\right)\right),\left(\mathrm{Weight}=1/\mathrm{y}\hat{\mkern6mu} 2\right) $$

x, Log [BD0801]; y, OD value.

### HUVEC proliferation inhibition assay

Inhibition of HUVEC proliferation was detected by CellTiter Glo staining (Promega, Madison, WI, USA). HUVECs in the logarithmic growth phase were plated into 96-well plates in ECM medium (ScienCell, #1001) containing 1% FBS (ScienCell, #0025) and 1% penicillin/streptomycin (GIBCO, #15140–122). After 18–20 h, different concentrations of BD0801 (Jiangsu Simcere Pharmaceutical Co., Ltd., Nanjing, China) or bevacizumab (Genentech/Roche, San Francisco, CA, USA) were incubated with 50 ng/ml VEGF (PrimeGene, Shanghai, China, #105–05) in ECM medium containing 0.5% FBS and 1% penicillin/streptomycin for 2 h at 37 °C before they were added into the HUVEC culture. The HUVECs were cultured at 37 °C, 5% CO_2_ for 72 h. CellTiter Glo working solution was added to the cells at room temperature according to the manufacturer’s manual. Then, the luminescence was measured by PheraStar FS (BMG Labtech, Offenburg, Germany). The IC_50_ was calculated based on the luminescence at different concentrations by GraphPad Prism 8.0 (GraphPad Software, San Diego, CA, USA). Each concentration was measured in triplicates in each experiment, and the experiment was repeated three times.

### HUVEC apoptosis assay

HUVECs in the logarithmic growth phase were plated into 24-well plates in ECM medium (ScienCell, #1001) containing 1% FBS (ScienCell, #0025) and 1% penicillin/streptomycin (GIBCO, #15140–122). After 18–20 h, different concentrations of BD0801 (Jiangsu Simcere Pharmaceutical Co., Ltd., Nanjing, China) or bevacizumab (Genentech/Roche, San Francisco, CA, USA) were incubated with 25 ng/ml VEGF (PrimeGene, Shanghai, China, #105–05) in ECM medium containing 0.5% FBS and 1% penicillin/streptomycin for 2 h at 37 °C before they were added into the HUVEC culture. The HUVECs were cultured at 37 °C, 5% CO_2_ for 48 h. The HUVECs were fixed by 4% paraformaldehyde and ethanol and stained by DAPI (Abcam, #Ab104139) to reveal the chromosome status. The apoptotic cells were identified as cells with altered nuclei, such as condensed chromosomes or disintegrated nuclei. Each condition was performed in triplicates.

### HUVEC cell cycle analysis

HUVECs in the logarithmic growth phase were plated into 6-well plates in ECM medium (ScienCell, #1001) containing 1% FBS (ScienCell, #0025) and 1% penicillin/streptomycin (GIBCO, #15140–122). After 18–20 h, different concentrations of BD0801 (Jiangsu Simcere Pharmaceutical Co., Ltd., Nanjing, China) or bevacizumab (Genentech/Roche, San Francisco, CA, USA) were incubated with 50 ng/ml VEGF (PrimeGene, Shanghai, China, #105–05) in ECM medium containing 0.5% FBS and 1% penicillin/streptomycin for 2 h at 37 °C before they were added into the HUVEC culture. The HUVECs were cultured at 37 °C, 5% CO_2_ for 48 h. The HUVECs were digested with 0.25% trypsin-EDTA (GIBCO, #25200–056), fixed by 75% ethanol, and stained by PI (Invitrogen, #P3566) to reveal the cell cycle distribution by flow cytometry analysis using BD FACSCanto II (BD, Franklin Lakes, NJ, USA).

### HUVEC migration inhibition assay

Cell migration assay was conducted using the Boyden Chamber Transwell method. The inner chamber with an 8.0-μm pores polycarbonate membrane was used. HUVECs suspended in HUVEC complete medium without serum were cultured in the inner chamber. Different concentrations of BD0801 (Jiangsu Simcere Pharmaceutical Co., Ltd., Nanjing, China) or bevacizumab (Genentech/Roche, San Francisco, CA, USA) were incubated with 20 ng/ml VEGF (Peprotech, Cranbury, NJ, USA, #100–20-10 μg) for 30 min at 37 °C before they were added into the outer chamber. The inner chamber was placed into the outer chamber. After incubation at 37 °C for 16 h, the medium in the inner chamber was removed, and the cells within the inner chamber were wiped off using cotton swabs. Then, the medium in the outer chamber was removed, and cells in the outer chamber were washed with PBS, fixed with ethanol, and washed with PBS again. The migrated cells were stained by crystal violet at room temperature for 20 min and washed with PBS to remove the residual dye. The effect of the test articles on the migration of HUVECs was observed under the microscope. Each condition was performed in triplicates.

### HUVEC scratch assay

HUVECs in the logarithmic growth phase were plated into 96-well plates and cultured overnight. The cells were starved in the standard medium without FBS at 37 °C for 16 h, and then wounds were created simultaneously in all wells. The cells were washed with PBS. Different concentrations of BD0801 (Jiangsu Simcere Pharmaceutical Co., Ltd., Nanjing, China) or bevacizumab (Genentech/Roche, San Francisco, CA, USA) were incubated with 20 ng/ml VEGF (Peprotech, Cranbury, NJ, USA, #100–20-10 μg) for 30 min at 37 °C before they were added into the wells. The plate was incubated at 37 °C, and the images of the wells were taken for 24 h. Each condition was performed in triplicates. The relative wound density was calculated as (Original wound area at 0 h-Wound area following treatment at different timepoint)/Original wound area at 0 h × 100%.

### Western blot

HUVECs were cultured in T25 culture flasks without FBS for 24 h. Different concentrations of BD0801 (Jiangsu Simcere Pharmaceutical Co., Ltd., Nanjing, China) or bevacizumab (Genentech/Roche, San Francisco, CA, USA) were incubated with 50 ng/ml VEGF (Peprotech, Cranbury, NJ, USA, #100–20-10 μg) for 30 min before they were added to HUVEC culture for 3 min. After incubation, the cells were harvested and lysed. The supernatant of cell lysate was heated at 100 °C for 10 min to be denatured and used in the SDS-PAGE, and the protein was transferred onto a PVDF membrane using a wet transfer system. The membrane was incubated with the primary antibody overnight, washed with Tris-buffered saline Tween (TBST), incubated with the secondary antibody for 1 h at room temperature, washed with TBST buffer, and then developed by ECL kit or luminescence. As for detailed antibody information, please see Supplementary Table S1. The Western blot analysis was repeated using the same protocol three times.

### Flow cytometry analysis (FACS)

For flow cytometry, tumors were disassociated with digestion enzyme mixture in GentleMACS Octo Dissociator with Heaters (Miltenyi Biotec, Bergisch Gladbach, Germany). The cells were suspended in FACS staining buffer as 1 × 10^6^ cells/100 μl in 96-well and stained with Fc Block for 5 min and other antibodies for 30 min, away from light at 4 °C. The cells were washed, fixed, and analyzed by FACS LSR Fortessa X20 (BD, Franklin Lakes, NJ, USA). Purified Rat Anti-Mouse CD16/CD32 (Mouse BD Fc Block™), FITC-CD45, PerCP-Cy5.5-CD4, and APC-CD8 antibodies were purchased from BD, and the Catalog numbers are 553,142, 553,080, 550,954, and 553,035, respectively. BV421-Live/Dead was purchased from Thermo Fisher (Catalog No. L34964). APC-Cy7-CD3 antibody was purchased from Biolegend (Catalog No. 100222).

### Immunohistochemistry (IHC) and immunofluorescence (IF) staining

The tumor samples harvested at the end of the study were fixed in Zn fixing buffer for about 36 h at room temperature, changed to ddH_2_O for 5 min, dehydrated, and embedded in paraffin using the Leica ASP300S system and EG 1150H + C system (Leica, Wetzlar, Germany). For double staining of PD-1 and CD8, the paraffin slides were stained with Leica BOND-III using the double staining program with rabbit anti-mouse CD8 (Abcam, Cambridge, United Kingdom, #ab237723) labeled by HRP (Bond Polymer Refine Detection, Leica, #DS9800) and rabbit anti-mouse PD-1 (Abcam, #ab214421) labeled by AP (Bond Polymer Refine Red Detection, Leica, #DS9390). The stained slides were scanned using Leica Aperio CS2. Six fields were viewed and analyzed for each slide/sample using ImageScope (Leica). The percentage of PD-1^+^ cells within the CD8^+^ cells was analyzed by ImageJ (NIH, Bethesda, MD, USA), and the average percentage of the six fields was calculated for each sample. For single staining of PD-L1, the paraffin slides were stained with Leica BOND-III using the DAB staining program with rabbit anti-mouse PD-L1 (Cell Signaling Technology, Boston, MA, USA, #64988 s) labeled by HRP (Bond Polymer Refine Detection, Leica, #DS9800). For single staining of CD31, the slides were dewaxed, and the endogenous peroxidase was quenched using 3% hydrogen peroxide. The slides were blocked, stained by CD31 primary antibody (Pharmingen, BD Biosciences, Franklin Lake, NJ, USA, #550274), HRP-conjugated secondary antibody, and DAB (Beijing Zhongshan Jinqiao Biotechnology Co., Ltd., Beijing, China, #PV-9004). The stained slides were scanned using Leica Aeprio VERSA, and the images were analyzed by HALO (Indica Labs, Albuquerque, NM, USA). The microvessels were counted based on CD31 staining, and the MVD was calculated by the total microvessel count divided by the tumor area.

For IF, the tumor samples were harvested and embedded in paraffin, as described in the above section. For staining of PD-1 and CD8 using IF, the paraffin slides were dewaxed with Leica Autostainer XL. EDTA was used as the antigen retrieval reagent. The slides were stained sequentially with rabbit anti-mouse CD8 (Abcam, Cambridge, United Kingdom, #ab237723), goat anti-rabbit IgG H&L Alexa Fluor 594 (Abcam, #ab150080), rabbit anti-mouse PD-1 (Abcam, #ab214421), goat anti-rabbit IgG H&L Alexa Fluor 488 (Abcam, #ab150081) and DAPI (Abcam, #ab104139). The stained slides were viewed by Nikon ECLIPSE Ni-U (Nikon, Tokyo, Japan). Five fields were viewed and analyzed for each slide/sample in red, green, and blue channels. The percentage of PD-1^+^ cells within the CD8^+^ cells was analyzed by ImageJ (NIH, Bethesda, MD, USA). The average percentage of the five fields was calculated for each sample.

### Animals

All experimental procedures involving animals and their care were conducted in conformity with the State Council Regulations for Laboratory Animal Management (Enacted in 1988) and were approved by the Institutional Animal Care and Use Committee of the WuXi AppTec, People’s Republic of China. Female Balb/c nude mice at 6–8 weeks of age were purchased from Shanghai Sippr-BK Laboratory Animal Co., Ltd. (Shanghai, China). Female C57BL/6 and Balb/c mice of 6–8 weeks of age were purchased from Shanghai Lingchang Biotechnology Co., Ltd. (Shanghai, China).

### PC9 xenograft mouse model

To establish the PC9 xenograft mouse model, 5 × 10^6^ PC9 cells per mouse were injected subcutaneously into female Balb/c nude mice’s right flank. As the average tumor volume reached 100–150 mm^3^, the mice were randomly assigned to experimental groups and treated with vehicle, AZD9291, or BD0801 (Jiangsu Simcere Pharmaceutical Co., Ltd., Nanjing, China), respectively.

### PK and efficacy studies in the PC9 mouse model

For the PK study, each dosing group contained nine mice, and three subgroups were formed for sampling at different time points. On day 0, a single dose of different concentrations of BD0801 was injected i.v. into PC9 tumor-bearing mice. The mice were sampled at various time points, and 40 μl serum was collected for each mouse at each time point.

For the efficacy study, tumor-bearing mice were treated with vehicle control, intravenously (i.v.) twice a week, AZD9291 (5 mg/kg dissolved in 0.5% methylcellulose and 0.1% Tween80 in water), orally every day, or different concentration of BD0801 (dissolved in saline), i.v. twice a week. Treatment started on day 0. The tumor volume and the bodyweight of the animals were measured twice a week. On day 24, just before the last dose of BD0801, mice treated with vehicle and BD0801 were sampled for drug exposure analysis. On day 27, mice from the vehicle- and BD0801-treated groups were sampled again before all of the animals were sacrificed. The long diameter (a) and the short diameter (b) of the tumor were measured using a caliper, and the tumor volume was calculated using the following formula: V = 0.5 x a x b^2^. T/C (%) = T_RTV_/C_RTV_ × 100%. RTV, relative tumor volume; RTV = V_t_/V_0_; V_0_ is the tumor volume of the animal when treatment starts; V_t_ is the tumor volume of the animal someday after treatment; T_RTV_: the mean RTV of the treatment group; C_RTV_: the mean RTV of the control group.

### Efficacy studies in the 3LL and CT26 syngeneic mouse models

To establish the 3LL syngeneic mouse model, 2 × 10^6^ 3LL cells per mouse were injected subcutaneously into female C57BL/6 mice’s right flank. To establish the CT26 syngeneic mouse model, 3 × 10^5^ CT26 cells per mouse were injected subcutaneously into female Balb/c mice’s right flank. When the average tumor volume reached 50–80 mm^3^, the mice were randomly assigned to each group according to the tumor volume and treated with BD0801, anti-PD-1 antibody (BioXcell, Lebanon, NH, USA, clone #RMP1–14, #BE0146), anti-PD-L1 antibody (BioXcell, Lebanon, NH, USA, clone #10F.9G2, #BE0101), or combinations. Antibodies were injected on the same day sequentially. The tumor volume and the bodyweight of the animals were measured three times a week. Tumor samples harvested at the end of the study were divided into two parts: one part was digested for flow cytometry analysis, and the other part was fixed in Zn fixing buffer for immunohistochemistry.

### Statistical analysis

The comparison between BD0801 and bevacizumab in the VEGF/VEGFR2 binding blocking assay and HUVEC proliferation inhibition assay was analyzed by t TEST. The statistics of HUVEC migration inhibition assay, HUVEC apoptosis assay, HUVEC Western blot quantification, FACS, IHC, and IF analysis were analyzed by one-way ANOVA followed by uncorrected Fisher’s LSD. The statistics of scratch assay and tumor volume were analyzed by two-way ANOVA followed by Tukey’s multiple comparisons test. All analyses were performed using GraphPad Prism 8.0 (GraphPad Software Inc., San Diego, CA, USA). *P*-values < 0.05 were considered statistically significant.

## Results

### BD0801 blocks the binding of VEGF/VEGFR2

**Fig. 1:**
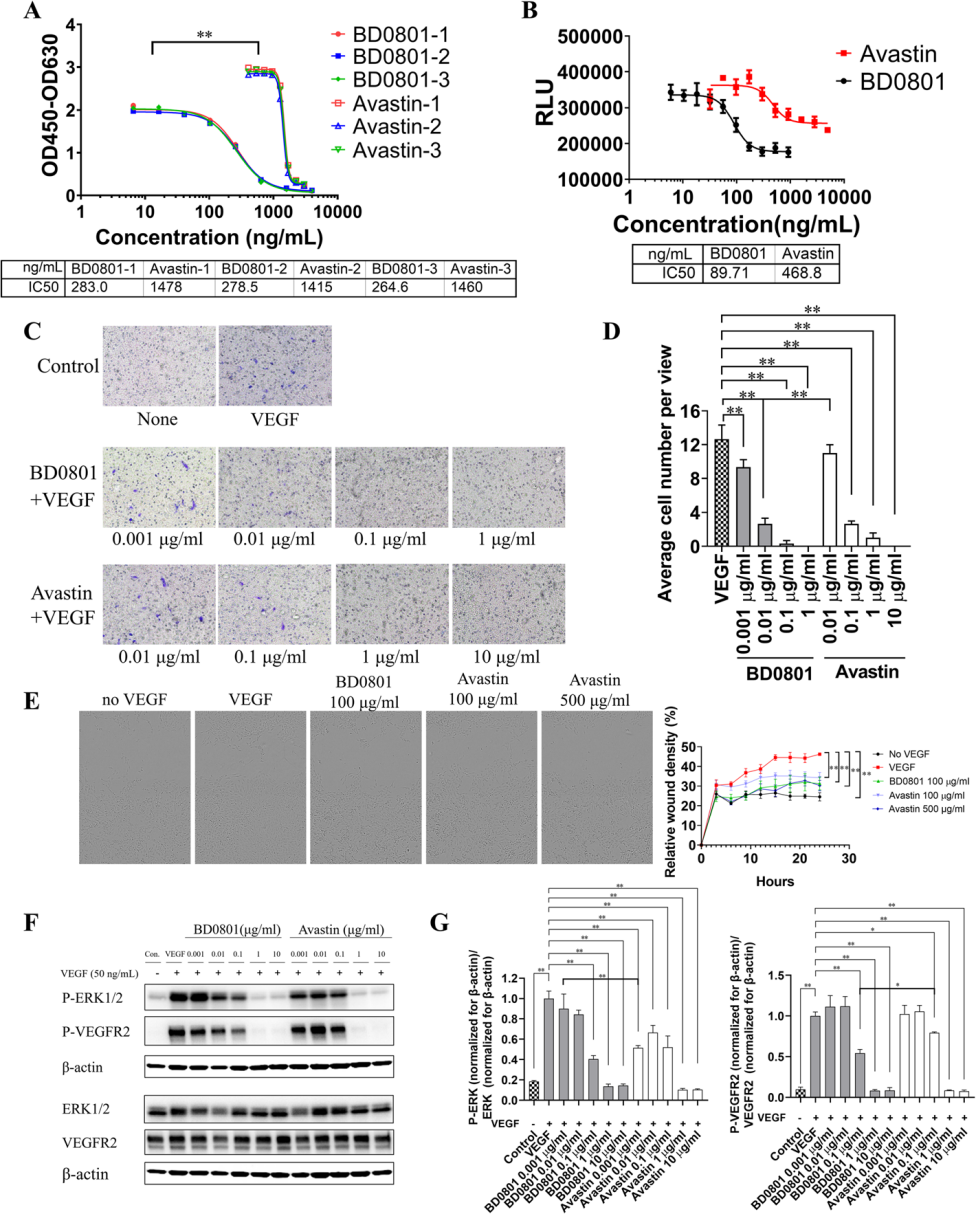
The effects of BD0801 on VEGF/VEGFR2 binding, downstream signaling, and HUVEC cellular functions in vitro. (A) Blockade of VEGF-VEGFR2 binding by BD0801 or bevacizumab (Avastin) was analyzed by ELISA. Results of three independent experiments. (B) Different concentrations of BD0801 or bevacizumab were incubated with 50 ng/ml VEGF for 2 h at 37 °C before they were added into the HUVEC culture. After 72 h, the inhibition of HUVEC proliferation was detected by CellTiter Glo staining. The experiment was performed three times, and one of the representative experiment results is shown. RLU, Relative light unit. (C) HUVECs were cultured in the inner chamber of the Boyden Chamber Transwell. Different concentrations of BD0801 or bevacizumab were incubated with 20 ng/ml VEGF for 30 min before adding them to the outer chamber. After incubating at 37 °C for 16 h, the migrated cells were stained purple by crystal violet and observed under the microscope. Each condition was performed in triplicates. (D) Quantification of HUVEC migration assay described in (C). (E) Different concentrations of BD0801 or bevacizumab were mixed and incubated with 20 ng/ml VEGF for 30 min before they were added to starved and scratched HUVEC culture. The relative wound densities were monitored and quantified for 24 h. The representative images at 24 h are shown. (F) Different concentrations of BD0801 or bevacizumab were mixed and incubated with 50 ng/ml VEGF for 30 min before they were added to HUVEC culture for 3 min. Protein extracts were separated by Western blot for phosphorylated-ERK1/2 (P-ERK1/2), phosphorylated-VEGFR2 (P-VEGFR2), ERK1/2, and VEGFR2. The three rows above (P-ERK1/2, P-VEGFR2, and β-actin) are from Blot 1; the three rows below (ERK1/2, VEGFR2, and β-actin) are from Blot 2. This Western blot analysis was repeated independently three times, and a representative result is shown. The original uncropped blots for this particular experiment are included in Supplementary S2. (G) The quantification and statistical analysis of the three independent Western blot experiments described in (F) are included. The value for VEGF only group was normalized to one. The quantification of P-ERK1/2, P-VEGFR2, ERK1/2, and VEGFR2 normalized for β-actin respectively are included in Supplementary Figs. S3 and S4. The error bars: standard error of the mean (SEM). *, *P* < 0.05, **, *P* < 0.01

Human VEGF was incubated with different concentrations of BD0801 (or the positive control bevacizumab) before the mixture was added to the ELISA plates (coated with VEGFR2-his) to determine the blocking effects of BD0801 on the binding of VEGF and VEGFR2. Both BD0801 and bevacizumab could effectively block VEGF binding to VEGFR2 with an IC_50_ of 275 ± 10 ng/ml and 1451 ± 32 ng/ml, respectively (Fig. 1A). BD0801 showed a significantly better blockade of VEGF/VEGFR2 binding than bevacizumab (*P* < 0.001).

### BD0801 inhibits the VEGF-mediated proliferation and migration of human umbilical vein endothelial cells (HUVECs)

BD0801 significantly inhibited the proliferation of HUVECs induced by VEGF. The IC_50_ was 87 ± 4 ng/ml, and the inhibition activity was significantly stronger than the positive control bevacizumab (IC_50_ = 476 ± 41 ng/ml, *P* < 0.01, Fig. 1B). In addition, the HUVECs apoptosis assay showed that both BD0801 and bevacizumab were able to induce apoptosis of HUVECs in the presence of VEGF. BD0801 at 0.1 μg/ml or above induced a significantly higher percentage of apoptotic HUVECs than bevacizumab at the same concentrations (*P* < 0.05, Supplementary Fig. S1A and S1B). Accordingly, the cell cycle analysis of the HUVECs also revealed that both BD0801 and bevacizumab were able to increase cells in the sub-G1 phase and reduce the cells in the G2/M phase dose-dependently (Supplementary Fig. S1C-E).

VEGF induced the migration of HUVECs, which was inhibited by BD0801 and bevacizumab. At 0.1 μg/ml, BD0801 nearly abolished the migration of HUVECs, and the potency of inhibition activity was 10-fold stronger than positive control bevacizumab (Fig. 1C-D). Similarly, BD0801 also showed more potent inhibition on wound healing activity of HUVEC cells than bevacizumab, demonstrated by a HUVEC scratch assay (Fig. 1E).

### BD0801 inhibits the VEGF-mediated activation of VEGFR2 and downstream signaling pathway in HUVECs

Considering the BD0801’s blocking effect in VEGF/VEGFR2 binding and its inhibition effect on proliferation and migration in HUVECs, we furthermore detected its potential underlying mechanism. VEGF was incubated with different concentrations of BD0801 or positive control bevacizumab. The mixture was then added to HUVEC cultures to investigate the effect of BD0801 on downstream signaling of VEGF in HUVECs. Here, we showed that VEGF significantly induced the phosphorylation of VEGFR2 and ERK1/2 in HUVECs (*P* < 0.01), and bevacizumab inhibited VEGF-mediated phosphorylation of VEGFR2 and ERK1/2. Similar to bevacizumab, BD0801 significantly inhibited both P-VEGFR2 and P-ERK1/2 levels in a dose-dependent manner (Fig. 1F-G, Supplementary Fig. S2–4).

### BD0801 inhibits the tumor growth in the lung cancer PC9 xenograft mouse model

**Fig. 2:**
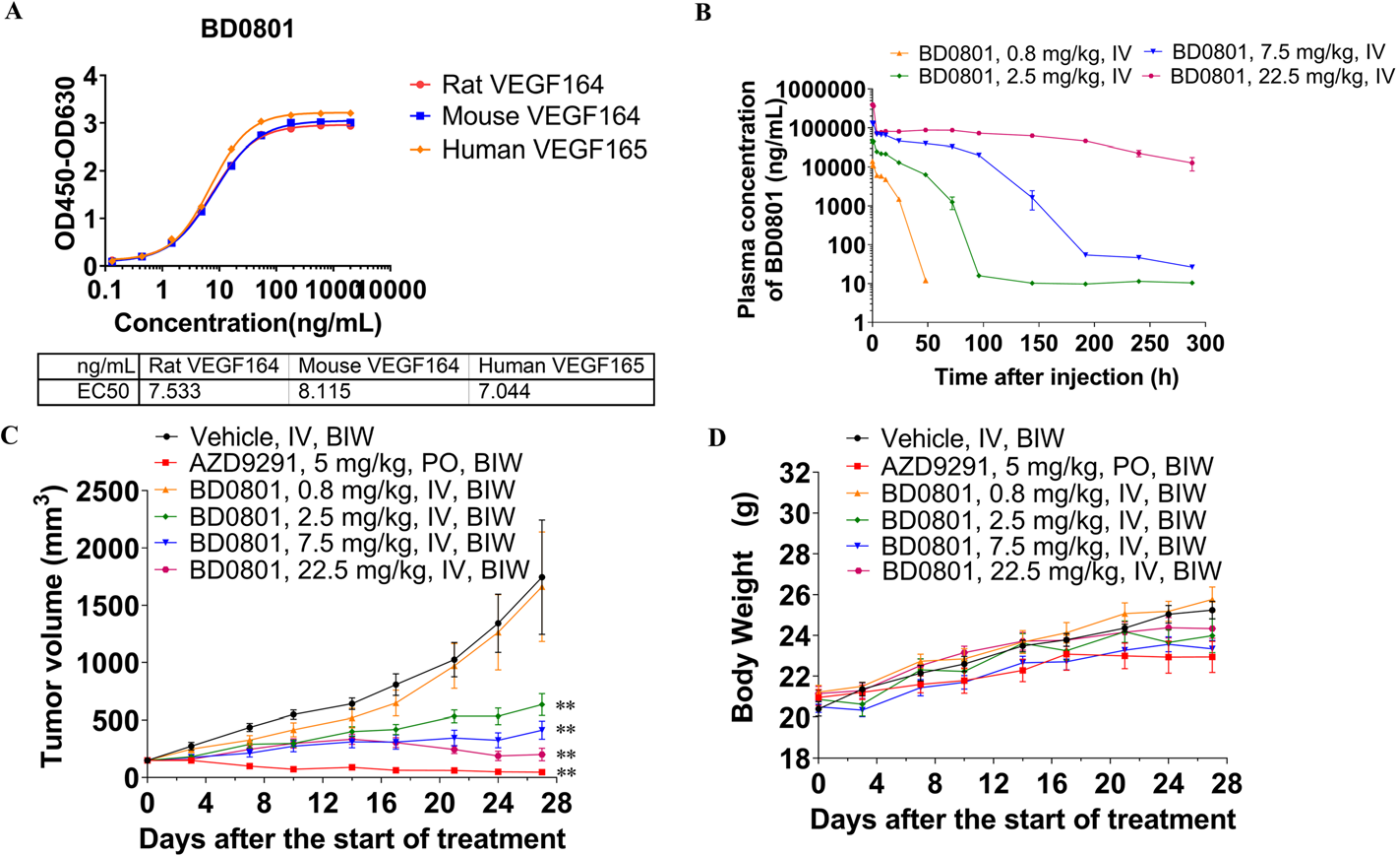
PK profile and antitumor effects of BD0801 in the PC9 lung cancer mouse models. (A) BD0801 has the same affinity for the rat, mouse, and human VEGF over the same concentration range. (B) PC9 cells were implanted subcutaneously into the right flank of Balb/c nude mice. When the average tumor volume reached ~ 150 mm^3^, the mice received a single intravenous injection of different concentrations of BD0801. Blood levels of BD0801 were analyzed by ELISA, *n* = 3/group. (C) Tumor volume of PC9 tumor-bearing Balb/c nude mice was administrated with the vehicle, AZD9291, or BD0801 biweekly (*n* = 6/group). (D) Bodyweight of PC9 tumor-bearing Balb/c nude mice was administrated with the vehicle, AZD9291, or BD0801 biweekly (n = 6/group). The error bars represent SEM. IV, intravenous; PO, oral gavage; BIW, biweekly. *P*-value was calculated based on the tumor volume comparing to the vehicle group using two-way ANOVA; **, *P* < 0.01. The tumor images are included in Supplementary Fig. S5

Since bevacizumab was used against lung cancer [9]; hence, lung cancer PC9 xenograft mouse models were used to examine the effect of BD0801. The PK profile of BD0801 in human lung cancer PC9 tumor-bearing Balb/c nude mice was first characterized, and the antitumor efficacy of BD0801 was then tested in this mouse model. As shown in Fig. 2A, BD0801 has the same affinity for rat, mouse, and human VEGF over the same concentration range, suggesting that it can be used reliably in mice and rats, and the results are translatable to humans.

As shown in Fig. 1B-D, 100 ng/ml BD0801 could significantly inhibit the proliferation and migration of HUVECs. When PC9 tumor-bearing Balb/c nude mice were given a single injection of 0.8 mg/kg BD0801, the serum concentration of BD0801 dropped below 100 ng/ml after 48 h. In contrast, animals treated with higher doses (2.5 mg/kg and above) resulted in higher serum (> 100 ng/ml) drug concentration, with the exposure maintained for at least 72 h (Fig. 2B). The data implied that in order to achieve a meaningful biological effect in this tumor model, a treatment regimen of no less than 2.5 mg/kg given at least every 3 days was required. Thus, the in vivo efficacy study was carried out with PC9 tumor-bearing mice treated with BD0801 using a twice-a-week dosing schedule for four weeks.

The PC9 xenograft mouse model responded well to the positive control AZD9291 (osimertinib), as expected. Based on the in vitro assay and PK data, the mice receiving 0.8 mg/kg BD0801 intravenously twice a week did not present a significant difference in tumor growth compared to the vehicle control. In contrast, mice receiving 2.5, 7.5, or 22.5 mg/kg BD0801 showed significantly reduced tumor growth. The ratios of treated tumor size relative to control (T/Cs) for the 2.5, 7.5, and 22.5 mg/kg BD0801 groups were 36.4, 23.7, and 11.6%, respectively, indicating a dose-dependent antitumor response of BD0801 (Fig. 2C). The serum concentrations of BD0801 of different groups on day 24 before the last injection of BD0801 and day 27 (72 h after the last injection) were measured. As expected, the C_trough_ of BD0801 in the 0.8 mg/kg was below 100 ng/ml, while the C_trough_ of all the other groups was above 100 ng/ml (Table 1). BD0801 was seen to be well tolerated without significant reduction of animal bodyweight (Fig. 2D).

**Table 1:** The concentration of BD0801 in the serum of PC9 tumor bearing mice

Group	Day 24 (ng/ml^1^)	Day 27 (ng/ml)
G1: Vehicle	BQL^2^	BQL
G3: BD0801 0.8 mg/kg	37 ± 8	28 ± 8
G4: BD0801 2.5 mg/kg	2325 ± 827	550 ± 281
G5: BD0801 7.5 mg/kg	102,282 ± 12,696	110,818 ± 14,784
G6: BD0801 22.5 mg/kg	447,714 ± 79,469	425,822 ± 88,948

Concentration data are shown as mean ± SD, *N* = 6

BQL: below the quantification limit

### BD0801 inhibits the tumor growth in the lung cancer 3LL syngeneic mouse model

**Fig. 3:**
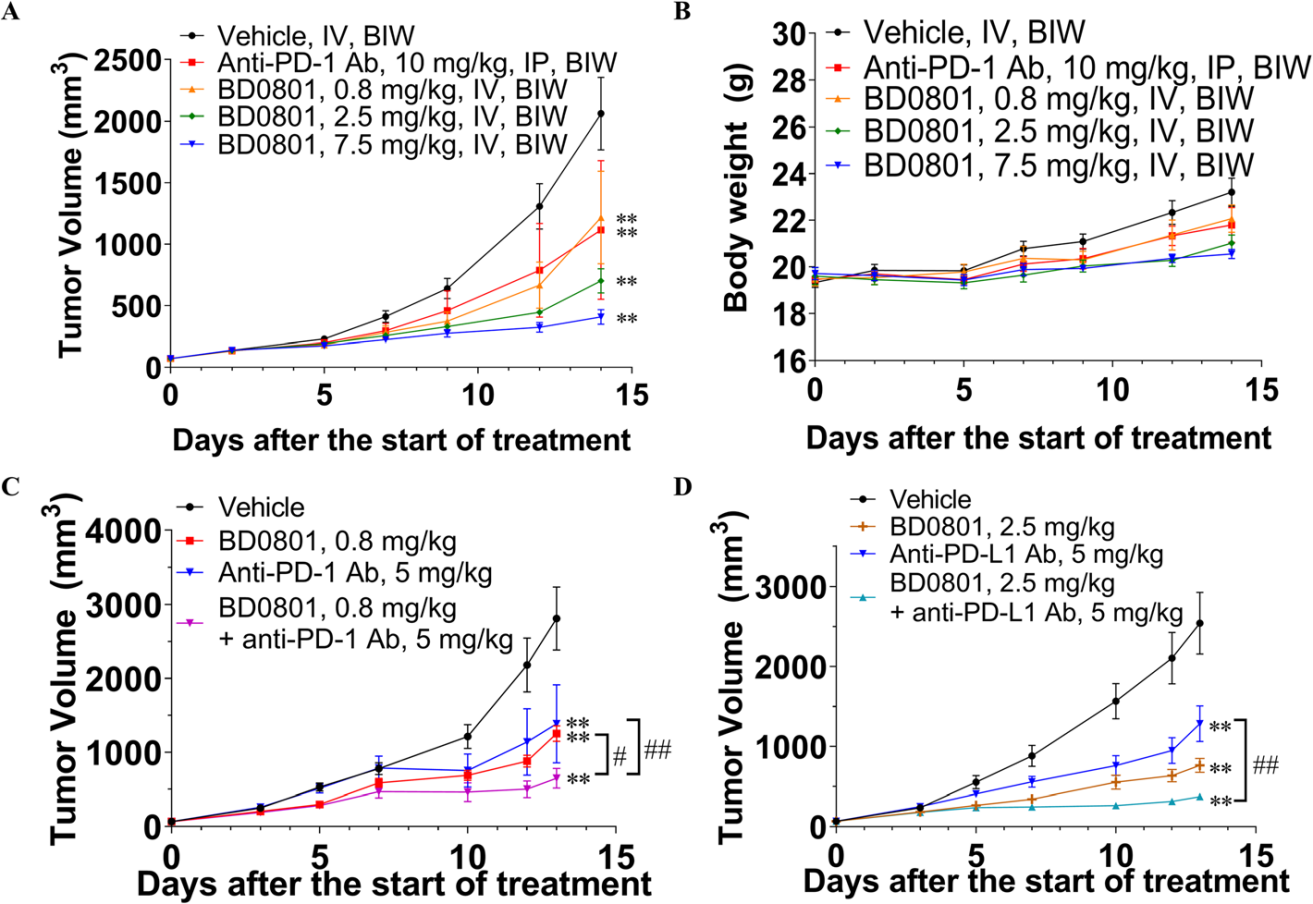
The antitumor effects of BD0801, anti-PD-1, and anti-PD-L1 antibodies in the 3LL lung cancer syngeneic mouse model. (A, B) The 3LL tumor-bearing C57BL/6 mice received the vehicle, anti-PD-1 antibody or BD0801 biweekly; the tumor volume and the bodyweight are shown in (A) and (B), respectively (n = 6/group). (C) The 3LL tumor-bearing C57BL/6 mice received 0.8 mg/kg BD0801, 5 mg/kg anti-PD-1 antibody, or combination by intraperitoneal injection biweekly (*n* = 10/group). (D) The 3LL tumor-bearing C57BL/6 mice received 2.5 mg/kg BD0801, 5 mg/kg anti-PD-L1 antibody, or combination by intraperitoneal injection biweekly (n = 10/group). The error bars represent SEM. IP, intraperitoneal, Ab, antibody. The P-value was calculated based on the tumor volume comparing to the assigned group using two-way ANOVA; treatment group versus vehicle group: **, P < 0.01; combination treatment group versus single treatment group: #, *P* < 0.05, ##, P < 0.01. The tumor images are included in Supplementary Fig. S5

A 3LL syngeneic mouse model was used to examine whether BD0801 can affect tumor growth in immunocompetent hosts. The dose-escalation efficacy study showed that BD0801 remarkably inhibited 3LL tumor growth in a dose-dependent manner, with T/Cs of 59.1, 34.0, and 19.8% for 0.8, 2.5, and 7.5 mg/kg BD0801, respectively (Fig. 3A). Neither anti-PD-1 antibody nor BD0801 reduced the animals’ bodyweight at all the dosages used (Fig. 3B).

### Combination of BD0801 with anti-PD-1 or anti-PD-L1 antibody exhibits synergistic tumor growth inhibition in a lung cancer model

Based on the single-agent efficacy study results in the 3LL model, the combination of BD0801 with anti-mouse PD-1 or anti-mouse PD-L1 antibody was assessed in the same model (Fig. 3C-D). Animals that received 0.8 or 2.5 mg/kg BD0801 with 5 mg/kg anti-PD-1 or 5 mg/kg anti-PD-L1 antibodies showed significantly reduced tumor growth compared to the vehicle group. The combination of 0.8 mg/kg BD0801 and 5 mg/kg anti-PD-1 antibody showed statistically better tumor growth inhibition compared to both single treatments (*P* < 0.05, T/C 44.4% for 0.8 mg/kg BD0801, T/C 54.2% for 5 mg/kg anti-PD-1 antibody, T/C 23.6% for combination, Fig. 3C). Similarly, the combination of 2.5 mg/kg BD0801 and 5 mg/kg anti-PD-L1 antibody also showed better tumor growth inhibition compared to both single treatments (T/C 30.6% for 2.5 mg/kg BD0801, T/C 53.2% for 5 mg/kg anti-PD-L1 antibody, T/C 15.1% for combination, Fig. 3D).

### The potential mechanism of the antitumor synergy between BD0801 and immunotherapy in combinatorial studies

The tumor microenvironment was analyzed at the end of the combinational efficacy study to investigate the mechanism of action behind the synergistic effects between BD0801 and ICIs. Upon termination of the combinatorial efficacy study using the 3LL tumor model (used for Fig. 3D), tumor samples were harvested. The tumors were analyzed for the percentage of CD4^+^ or CD8^+^ T cells by flow cytometry and the percentage of PD-1^+^ cells within CD8^+^ T cells and microvessel density (MVD) by immunohistochemistry.

**Fig. 4:**
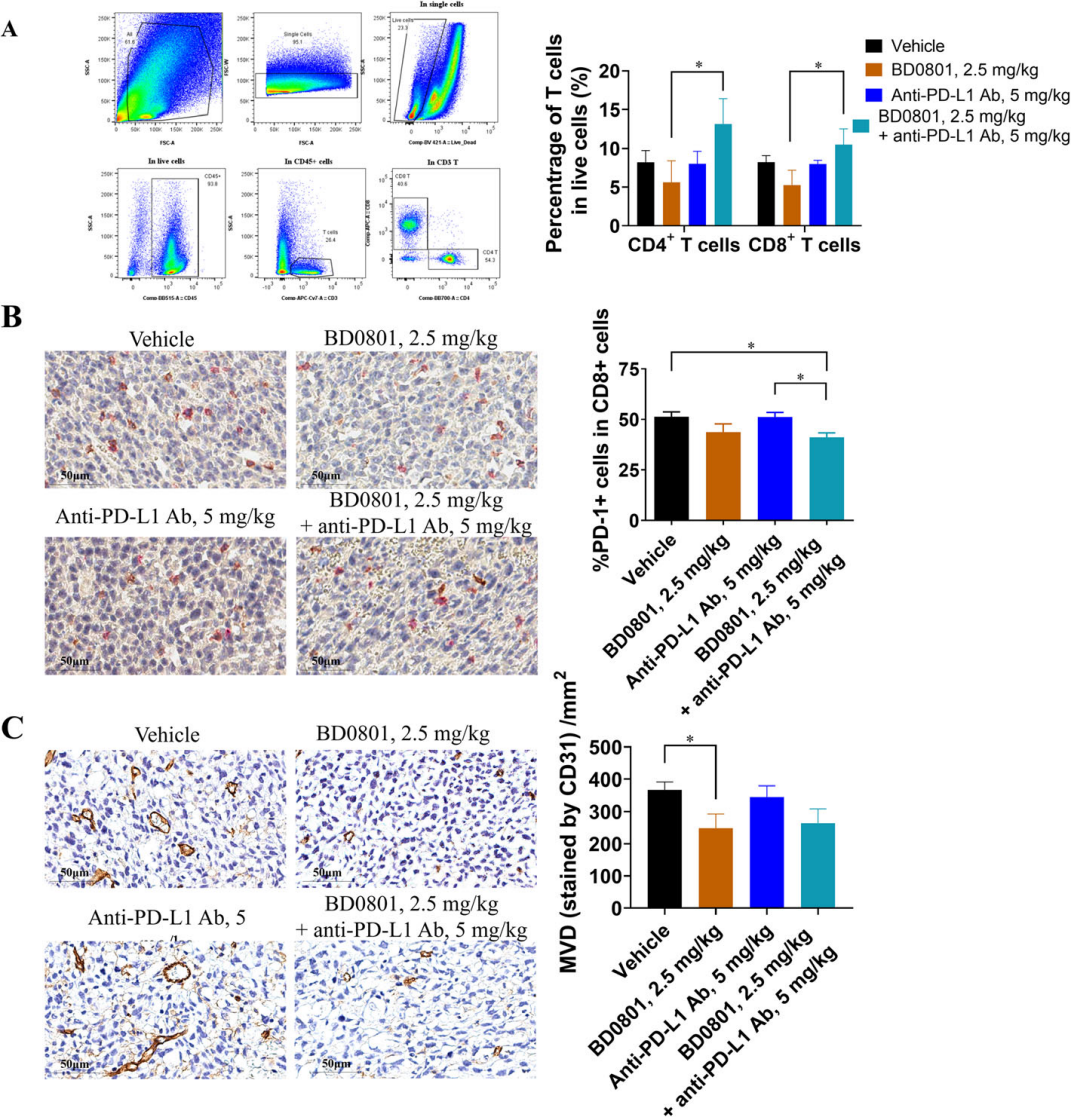
Ex vivo analysis of tumor samples from the 3LL syngeneic mouse model. (A) Left panel, representative FACS plots and gating strategy. Right panel, bar graphs of tumor-infiltrated T cells analyzed using FACS. Percentages of CD4^+^ T cells (defined as Live/Dead^−^CD45^+^CD3^+^CD4^+^) and CD8^+^ T cells (defined as Live/Dead^−^CD45^+^CD3^+^CD8^+^) in total live cells (defined as Live/Dead^−^) (n = 6/group). The representative flow images and gating strategy of 4 different groups are shown in Supplementary Fig. S7. (B) The representative images of the IHC double staining using anti-PD-1 and CD8 antibodies are shown, Red: PD-1; Brown: CD8. (left panel, scale bar: 50 μm). The average percentages of the double-positive staining of PD-1^+^CD8^+^ T cells were analyzed (right panel, n = 10/group). (C) The representative images of the IHC staining using anti-CD31 antibody are shown (left panel, scale bar: 50 μm). The CD31 IHC data were quantified (right panel, n = 6 for each group). The error bars represent SEM; *, P < 0.05

Upon 2.5 mg/kg BD0801, the percentages of CD4^+^ and CD8^+^ cells within live cells were slightly decreased (from 8.23 to 5.62% and 8.24 to 5.25%, respectively). Treatment with 5 mg/kg anti-PD-L1 antibody did not affect T cell percentage. In contrast, the combination of BD0801 and anti-PD-L1 antibody increased the percentage of tumor-infiltrated CD4^+^ and CD8^+^ cells compared to vehicle or single treatment groups (Fig. 4A). Based on the double immunohistochemistry staining of PD-1 and CD8, the percentage of PD-1^+^ cells within CD8^+^ T cells in the vehicle group was 51.35%. Upon 2.5 mg/kg BD0801 treatment, the percentage decreased to 43.70% without statistical significance, while treatment of 5 mg/kg anti-PD-L1 antibody did not affect the percentage. The combination of BD0801 and anti-PD-L1 antibody significantly decreased the percentage of CD8^+^/PD-1^+^ cells to 41.13% compared to vehicle and anti-PD-L1 antibody single treatment (Fig. 4B). This decreased percentage of CD8^+^/PD-1^+^ cells suggests that the combinatorial treatment reduced the subpopulation of tumor-infiltrated CD8^+^ cells with exhausting marker PD-1. Two-color immunofluorescence staining was performed to confirm the CD8^+^/PD-1 double-positive staining results, which generated similar results (Supplementary Fig. S6A and S6B). In addition, these tumor tissues were found to be relatively high PDL-1 expressers by IHC (Supplementary Fig. S6C). Based on the immunohistochemistry staining of CD31, MVD significantly decreased from 368/mm^2^ to 248/mm^2^ upon 2.5 mg/kg BD0801 treatment, while 5 mg/kg anti-PD-L1 antibody did not affect MVD (345/mm^2^). BD0801 and anti-PD-L1 antibody treatment combination decreased the MVD to 264/mm^2^, similar to BD0801 single treatment (Fig. 4C).

Taken together, the synergistic in vivo activities observed between BD0801 and anti-PD-L1 antibody might be related to the increased infiltration of CD4^+^ and CD8^+^ T cells, the decreased exhausted PD-1^+^ CD8^+^ T cells, and the reduction of vascularization in tumors.

### Combination of BD0801 with anti-PD-1 or anti-PD-L1 antibody exhibits synergistic tumor growth inhibition in a colorectal cancer model

**Fig. 5:**
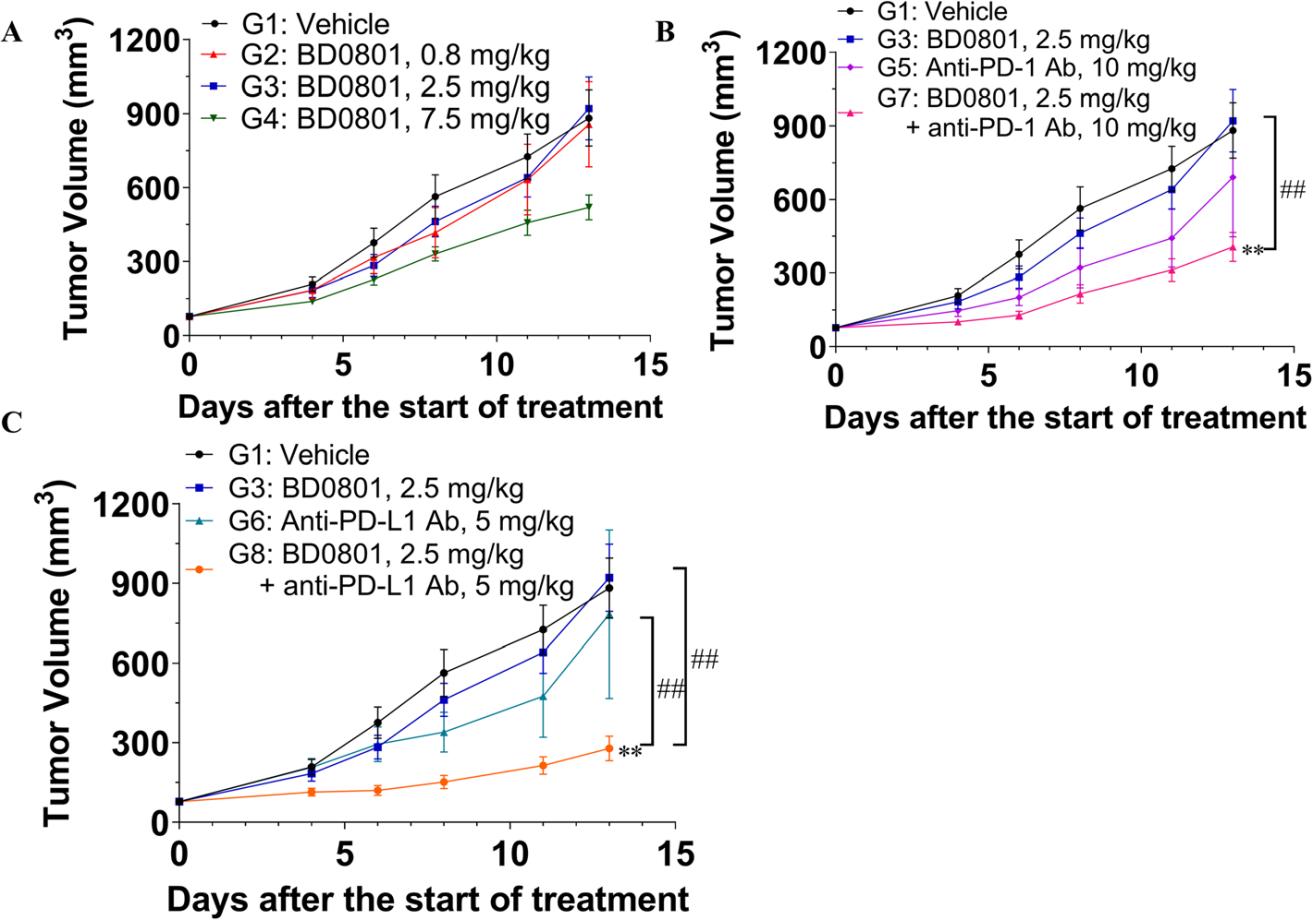
The antitumor effects of BD0801, anti-PD-1 antibody, and anti-PD-L1 antibody in CT26 colorectal cancer syngeneic mouse model. (A-C) The CT26 tumor-bearing Balb/c mice received BD0801, anti-PD-1 antibody, anti-PD-L1 antibody, or different combinations by intraperitoneal injection biweekly (n = 10/group). G, Group. The error bars represent SEM. The P-value was calculated based on the tumor volume comparing to the assigned group using two-way ANOVA; treatment group versus vehicle group: **, P < 0.01; combination treatment group versus single treatment group: ##, P < 0.01. The tumor images are included in Supplementary Fig. S5

Next, since bevacizumab and immunotherapy can be used in colorectal cancer [9, 13], the synergy of BD0801 and immunotherapy was tested in the mouse colorectal cancer synergistic model CT26 (Fig. 5). In this mouse model, 0.8 mg/kg and 2.5 mg/kg BD0801 did not inhibit tumor growth, with T/Cs of 94.7 and 106.9%, respectively. Only 7.5 mg/kg BD0801 affected the tumor growth, with a T/C of 64.7%, but was not statistically significant (Fig. 5A). Neither 10 mg/kg anti-PD-1 antibody nor 5 mg/kg PD-L1 antibody significantly affected tumor growth, with T/Cs of 87.8 and 112.7%, respectively. In contrast, the combination of 2.5 mg/kg BD0801 and 10 mg/kg anti-PD-1 antibody significantly reduced tumor growth, with a T/C of 46.8% (Fig. 5B), as well as the combination of 2.5 mg/kg BD0801 and 5 mg/kg anti-PD-L1 antibody, with a T/C of 31.3% (Fig. 5C). The latter combination also showed statistically significant antitumor synergy compared to single-agent treatment groups (*P* < 0.01).

## Discussion

The VEGF/VEGFR network is often upregulated in tumor cells and tumor microenvironment [6]. Signaling through VEGF and its receptor plays critical roles in promoting cancer cell proliferation and angiogenesis and supporting an immuno-suppressive tumor microenvironment [1, 5–7, 22, 23]. The present study was designed to determine the effectiveness of an anti-VEGF monoclonal antibody, BD0801, combined with anti-PD-1/PD-L1 antibodies in preclinical tumor models to elucidate the mechanisms and support combination strategies in future clinical studies.

This study first demonstrated the effectiveness of the anti-VEGF monoclonal humanized antibody BD0801 using biochemical and cellular assays. We show that BD0801 targets VEGF/VEGFR-mediated angiogenesis. Notably, as assessed by the in vitro assays, including VEGF/VEGFR binding blockade and VEGF-activated HUVECs proliferation/migration inhibition, BD0801 has a 5–10-fold higher potency than bevacizumab. Since the binding affinity of BD0801 and bevacizumab to human VEGF is similar (unpublished data), the greater potency of BD0801 in the in vitro functional assay may come from the different binding epitopes to VEGF. These results are supported by Liu et al. [30], who reported that BD0801 could inhibit the growth and induce the apoptosis of HCC cells, and by Zhang et al. [31], who showed that BD0801 decreased tumor volume in models of colorectal tumors. BD0801 has also been used in various in vitro studies that suggest its anti-VEGF efficacy [32–35].

The ORRs of ICIs alone are only 10–40% in solid tumors [19], and combination strategies could be the key to improve their efficacy [12, 20, 21]. Indeed, immune escape is a major mechanism of tumor survival and resistance to treatments. Therefore, attacking the tumor from multiple fronts simultaneously, including immune escape, could be an efficient treatment strategy [12, 20, 21]. In addition to the well-known involvement of PD-1/PD-L1 in the immune escape, VEGF/VEGFR signaling can affect immune cells by increasing Tregs, suppressing the proliferation of CD8^+^ T cells, and increasing the expression of PD-1, TIM-3, LAG-3, and CTLA-4 [22–24]. Hence, concepts of combining an anti-VEGF treatment with an anti-PD-1/PD-L1 drug are appealing. In this study, we first determined that a biweekly 2.5-mg/kg treatment of BD0801 resulted in sufficient drug serum (> 100 ng/ml) concentration which could produce significant antitumor growth activities in vivo. The subsequent efficacy experiments showed that combining BD0801 with anti-PD-1/PD-L1 drugs indeed potentialized the antitumor effect in lung and colorectal cancer models. These results are supported by the atezolizumab and bevacizumab combination in NSCLC [29]. They are the basis for ongoing clinical trials in renal cancer and HCC (ClinicalTrials.gov #NCT01984242 and #NCT02715531).

The potential mechanisms underlying the antitumor synergy were explored. This study showed that animals in the combination groups had a consistent trend of increased infiltrated CD4^+^ and CD8^+^ T cells, significantly decreased percentage of double-positive PD-1^+^ CD8^+^ T cells, and reduced MVD in tumor tissues. This indicates that activated T-cell-mediated immunity and compromised angiogenic activity might contribute to the synergistic antitumor efficacy. These results are supported by the studies discussed above [12, 20–24]. Interestingly, a low dose of BD0801 (0.8 mg/kg) could synergize with anti-PD-1 therapy, suggesting that a lower dose of BD0801 might be used when combined with immunotherapy in clinical studies.

Several lines of evidence have shown that antiangiogenic agents can decrease immunosuppressive cells, immunosuppressive cytokines, and inhibitory molecules on T cells to reverse the immunosuppression [36, 37], suggesting a rationale to combine antiangiogenic agents with immune checkpoint blockade. Two studies showed that the combination of anti-mouse VEGF antibody or anti-mouse VEGFR2 antibody with anti-mouse PD-1 antibody presents synergetic antitumor effects in the colorectal mouse model [24, 27]. In one of these studies, upon treatment with the anti-VEGF antibody, there was a reduction of the percentage of PD-1^+^/TIM-3^+^ cells within CD8^+^ T cells, similar to the present study [24]. In the other study, there was an increase of CD4^+^ T cells with a reduction of MVD in tumors of animals treated with the combination of anti-VEGFR2 and anti-PD-1 antibodies compared to the anti-VEGFR2 single treatment group, which also support the present study [24, 27]. A recent study showed that the addition of anti-VEGF therapy to an anti-PD-L1 regimen could synergistically improve the outcome of the anti-PD-L1 treatment by using an autochthonous mouse model of SCLC, a model that is less sensitive to anti-PD-L1 treatment [28]. Interestingly, the investigators also found that anti-PD-L1 monotherapy increased the percentage of PD-1^+^/TIM-3^+^/CD8^+^ exhausted T cells in the tumor. Still, the combinational treatment completely reversed T cells’ exhaustion, leading to significantly enhanced antitumor activity [28]. In the present study, no significant increase of exhausted T cells was observed with anti-PD-L1 alone. This might be because the 3LL tumor model is sensitive to anti-PD-1/PD-L1 therapy or due to different time points at which the samples were harvested between the two studies. Future studies are still necessary to refine the results.

Vasculature normalization has been proposed to play an important role in cancer therapies involving antiangiogenic agents. Interestingly, a study combining an anti-VEGFR2 antibody and cancer cell vaccine in a breast cancer mouse model showed that a lower dose of anti-VEGFR2 antibody could normalize the tumor vasculature, modulate the immunosuppressive tumor microenvironment, and synergize with cancer cell vaccine, while a full dose of anti-VEGFR2 could not [38]. In this study, a consistent trend of reduced MVD was observed upon BD0801 treatment. Still, no additional MVD reduction was observed in the combination group compared to the single agent-treated groups. While these results are somewhat puzzling, we propose that the vasculature normalization induced by BD0801 increases the accessibility of both immune cells and therapeutic antibodies within the tumor, which in turn facilitates antitumor synergy with immunotherapy [39, 40].

This study has limitations. Intervention-induced antiangiogenesis and vasculature normalization is a dynamic process, and only the tumor tissues collected at the end of the study were analyzed, which may not reflect a complete picture of the pharmacodynamics modulation. Further studies characterizing the tumor vasculature normalization effects of BD0801 using more sophisticated assays at different time points may shed more light on these mechanisms.

## Conclusions

In conclusion, this study demonstrates BD0801 as an effective antiangiogenic and antitumor agent in vitro and in vivo. Notably, the combination of BD0801 with an ICI reveals synergistic antitumor effects in both lung cancer and colorectal cancer mouse models. The mechanism of action underlying this antitumor synergy between BD0801 and ICIs might involve enhanced T-cell mediated immunity and possibly improved vasculature normalization in the tumor microenvironment. These data suggest that BD0801 might be a promising antiangiogenic agent with a clinical development value combined with cancer immunotherapy. Based on this study and published data, current future directions include conducting clinical trials evaluating BD0801 alone and in combination.

## Supplementary Information

**Additional file 1.**

## Data Availability

The data set supporting the results of this article are included within the article. The datasets used and/or analyzed during the current study are available from the corresponding author on reasonable request.

## Abbreviations

CTLA-4: cytotoxic T lymphocyte antigen 4
FDA: Food & Drug Administration
HCC: hepatocellular carcinoma
HUVECs: human umbilical vein endothelial cells
ICIs: immune checkpoint inhibitors
LAG-3: lymphocyte activation gene-3
MDSCs: myeloid-derived suppressor cells
MOA: mechanisms of actions
MVD: microvessel density
NSCLC: non-small cell lung cancer
ORRs: objective response rates
PD-1: programmed cell death protein 1
PD-L1: programmed cell death ligand 1
TIM-3: T-cell immunoglobulin mucin 3
Treg: regulatory T
VEGF: vascular endothelial growth factor
