# Supplementary material for: Antiangiogenic antibody BD0801 combined with immune checkpoint inhibitors achieves synergistic antitumor activity and affects the tumor microenvironment
Source: BMC Cancer. 2021 Oct 22;21:1134. doi: 10.1186/s12885-021-08859-5 (PMC8539826; doi:10.1186/s12885-021-08859-5)
Supplement: Supplementary file 1 — Additional file 1. [file 12885_2021_8859_MOESM1_ESM.docx]

**Supplementary Materials for**

**Antiangiogenic antibody BD0801 combined with immune checkpoint inhibitors achieves synergistic antitumor activity and affects the tumor microenvironment**

**Running title:** Synergistic antitumor activity of BD0801+ICI

Liting Xue^a#^, Xingyuan Gao^a#^, Haoyu Zhang^a^, Janxing Tang^a^，Qian Wang^a^, Feng Li^b^, Xinxin Li^a^, Xiaohong Yu^a^, Zhihong Lu^c^, Yue Huang^a^, Renhong Tang^a^ and Wenqing Yang^a^*

^a^State Key Laboratory of Translational Medicine and Innovative Drug Development, Jiangsu Simcere Pharmaceutical Co. Ltd., Nanjing Jiangsu, China

^b^DMPK and Clinical Pharmacology, Suzhou Ribo Life Science Co. Ltd., Kushan Jiangsu, China

^c^Green Valley Research Institute, Shanghai Green Valley Pharmaceutical Co., Ltd., Shanghai, China

#These two authors contributed equally to this manuscript.

***Corresponding Author:** WenQing Yang

Jiangsu Simcere Pharmaceutical Co. Ltd, Xuanwu Road 699-18, Xuanwu District, Nanjing, Jiangsu, China

**Phone Numbers:** +86-18551211356

**Email Address:** wenqing.yang@cn.simcere.com

**Supplementary Table S1**

| antibody | Company | dilution | Source | Cat. |
| --- | --- | --- | --- | --- |
| p44/42 MAPK(ERK1/2) (137F5） | CST | 1:1000 | Rabbit mAb | 4695 |
| MAPK phospho p44/42 (T202/Y204) | CST | 1:1000 | Rabbit mAb | 4370 |
| VEGFR2 | CST | 1:1000 | Rabbit mAb | 2479S |
| Phospho-VEGF Receptor 2 (Tyr1175) (19A10) Rabbit mAb | CST | 1:1000 | Rabbit mAb | 2478S |
| β-actin | CST | 1:2000 | Mouse mAb | 3700S |
| Anti-rabbit IgG, HRP-linked Ab | CST | 1:1000 | Goat | 7074 |
| IRDyeⓇ 680RD Goat anti-Mouse IgG (H+L) | Licor | 1:5000 | Goat | 926-68070 |

Note: CST, Cell Signaling Technology, Boston, MA, USA; Licor, Lincoln, NE, USA

**Supplementary Figure S1**

**A**

**
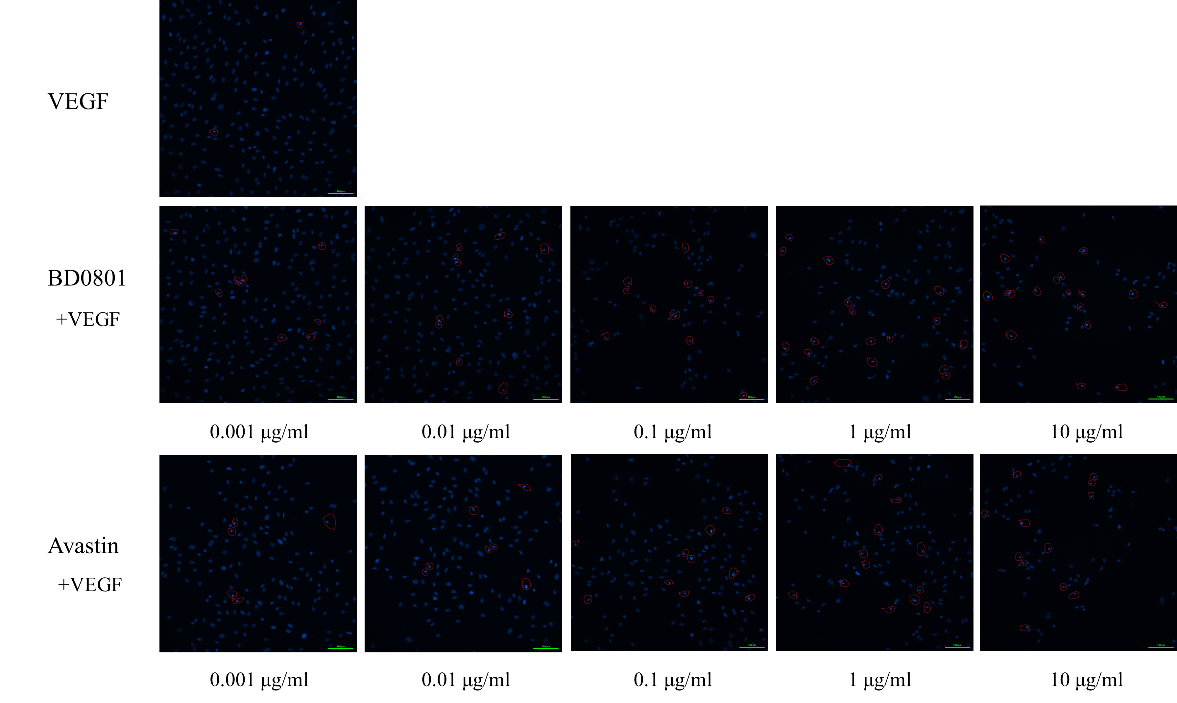
**

**B**

**
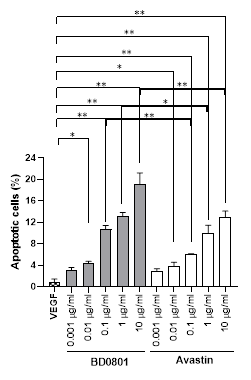
**

**C**

**
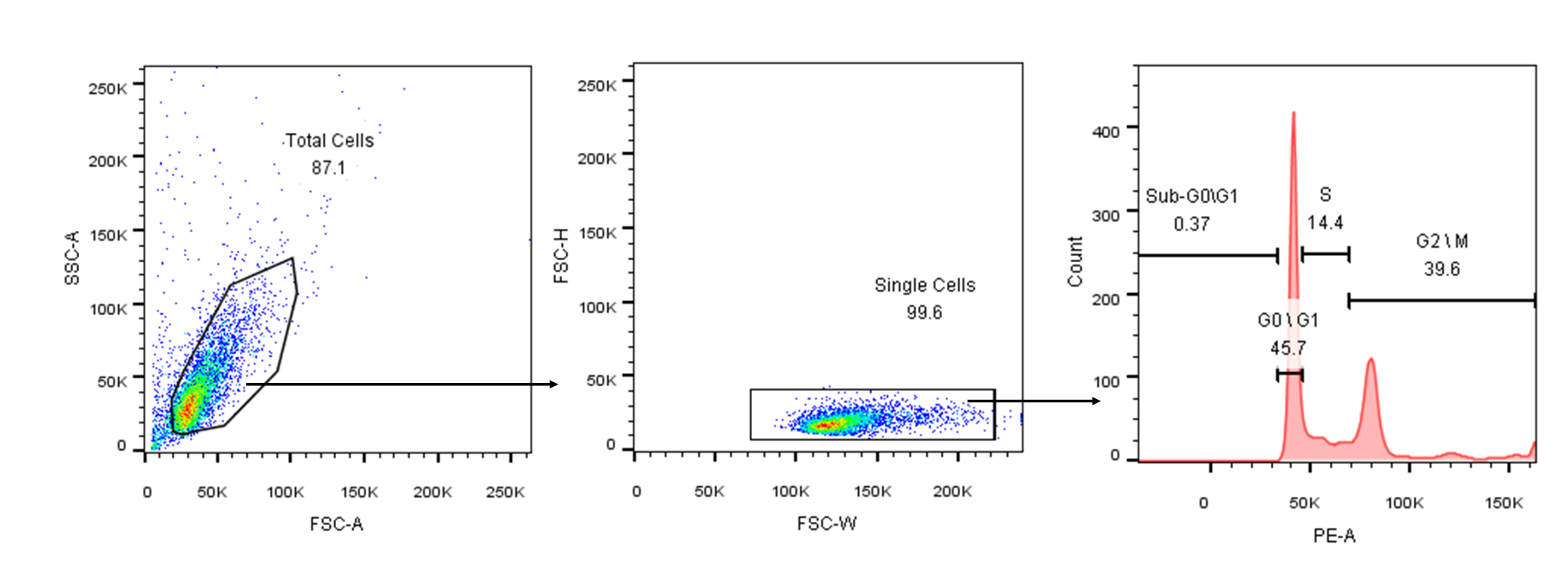
**

**D**

**
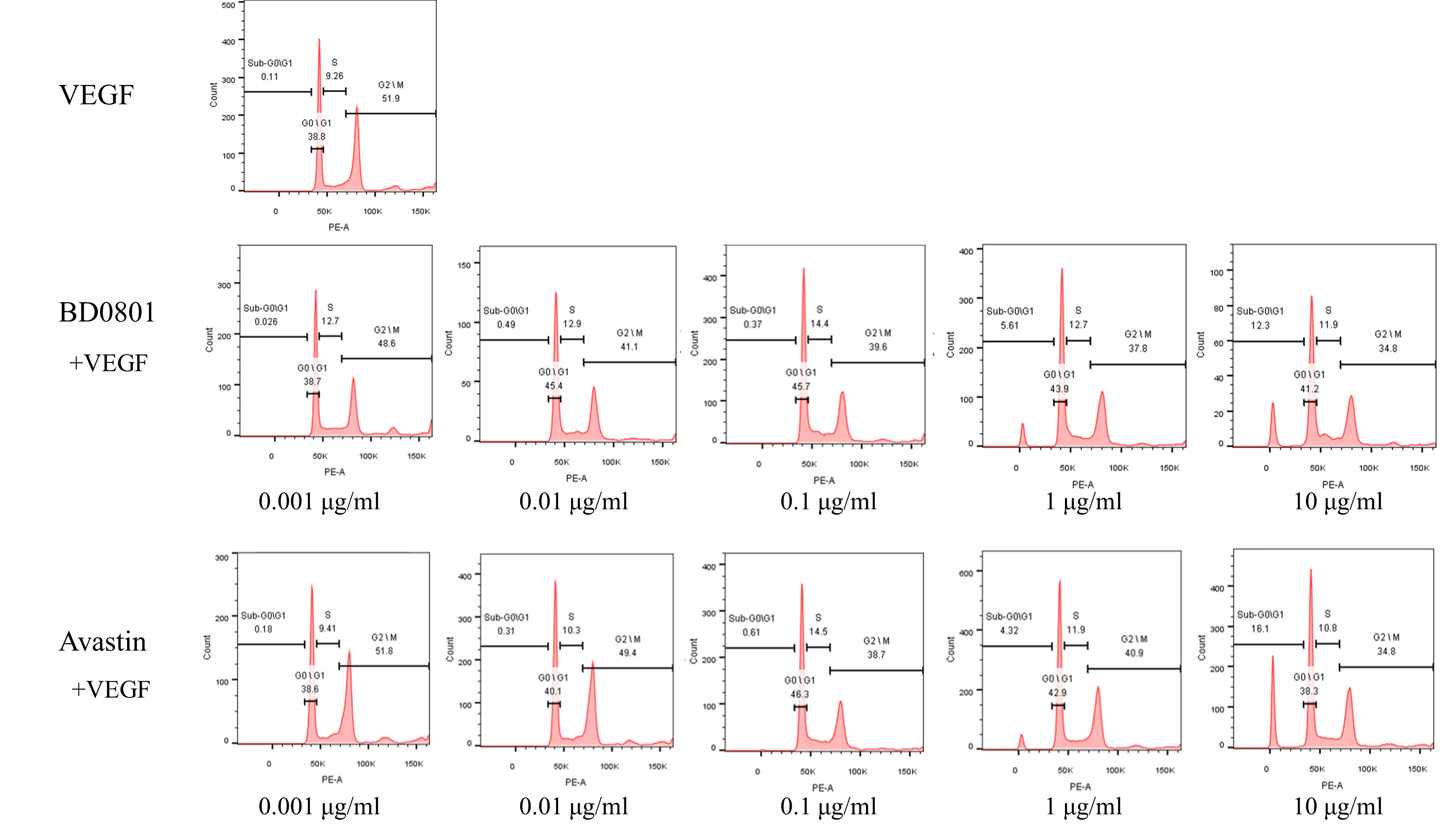
**

**E**

**Supplementary Figure S1. HUVEC apoptosis and cell cycle assay.** (A, B) Different concentrations of BD0801 or bevacizumab were incubated with 25 ng/ml VEGF for 2 h at 37^o^C before they were added into the HUVEC culture. After 48 h, the apoptosis of HUVEC was detected by DAPI staining, and the representative images are shown (A) (scale bar: 100 μm). The apoptotic cells were indicated by red circles. Quantification of the HUVEC apoptosis assay is shown in (B). (C-E) Different concentrations of BD0801 or bevacizumab were incubated with 50 ng/ml VEGF for 2 h at 37^o^C before they were added into the HUVEC culture. After 48 h, the cell cycle status of HUVEC was detected by PI staining using flow cytometry analysis. The gating strategy is shown in (C). The FACS plots are shown in (D). The quantification of the cell cycle distribution is shown in (E). The error bars represent SEM; *, P<0.05, **, P<0.01.

**Supplementary Figure S2**

**Blot 1:**

P-ERK1/2


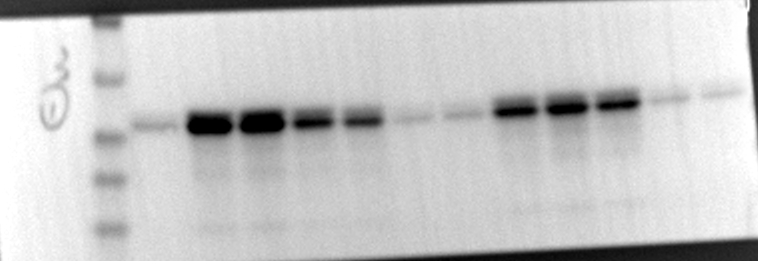


P-VEGFR2


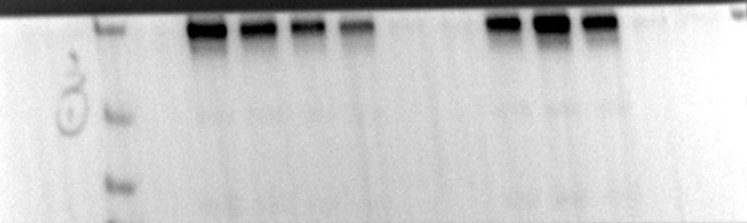


β-actin (loading control)





**Blot 2:**

ERK1/2


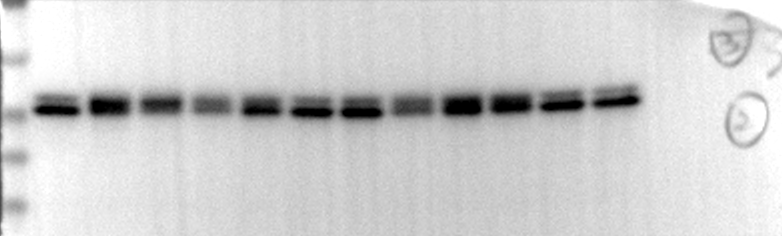


VEGFR2


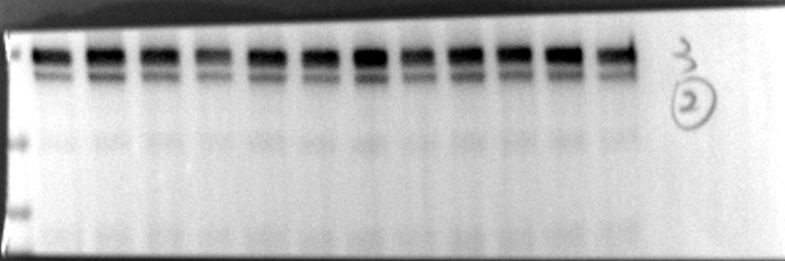


β-actin (loading control)





**Supplementary Figure S2. Original uncropped blots for Figure 1F.** Blot 1 shows the images of the original uncropped blots for three above rows in Figure 1F, and blot 2 shows the images of the original uncropped blots for three below rows in Figure 1F.

**Supplementary Figure S3**

**A**

**B**

**Supplementary Figure S3. Quantification of P-ERK1/2 and P-VEGFR2 normalized for the expression of β-actin for Figure 1F.** The Western blot analysis for P-ERK1/2, P-VEGFR2, ERK1/2, and VEGFR2 in HUVECs treated with BD0801 or bevacizumab was repeated three times. One representative result is shown in Figure 1F and the quantification analysis of P-ERK1/2 and P-VEGFR2 normalized for β-actin in the three independent experiments are shown above. The error bars represent SEM.

**Supplementary Figure S4**

**A**

**B**

**Supplementary Figure S4. Quantification of ERK1/2 and VEGFR2 normalized for the expression of β-actin for Figure 1F.** The Western blot analysis for P-ERK1/2, P-VEGFR2, ERK1/2, and VEGFR2 in HUVECs treated with BD0801 or bevacizumab was repeated three times. One representative result is shown in Figure 1F and the quantification analysis of ERK1/2 and VEGFR2 normalized for β-actin in the three independent experiments are shown above. The error bars represent SEM.

**Supplementary Figure S5**

Tumor images for Figure 2C

| Vehicle | **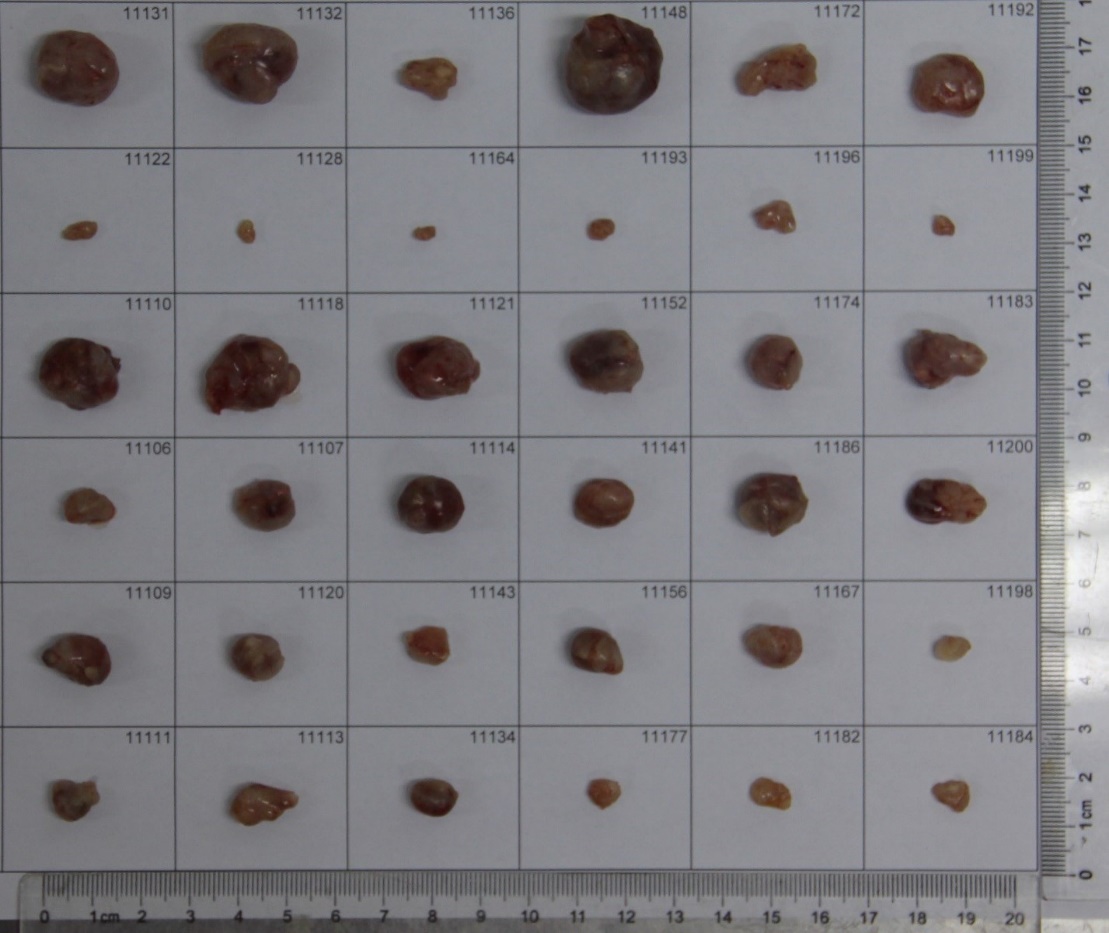** |
| --- | --- |
| AZD9291  5 mg/kg |  |
| BD0801  0.8 mg/kg |  |
| BD0801  2.5 mg/kg |  |
| BD0801  7.5 mg/kg |  |
| BD0801  22.5 mg/kg |  |

Tumor images for Figure 3A

| Vehicle | **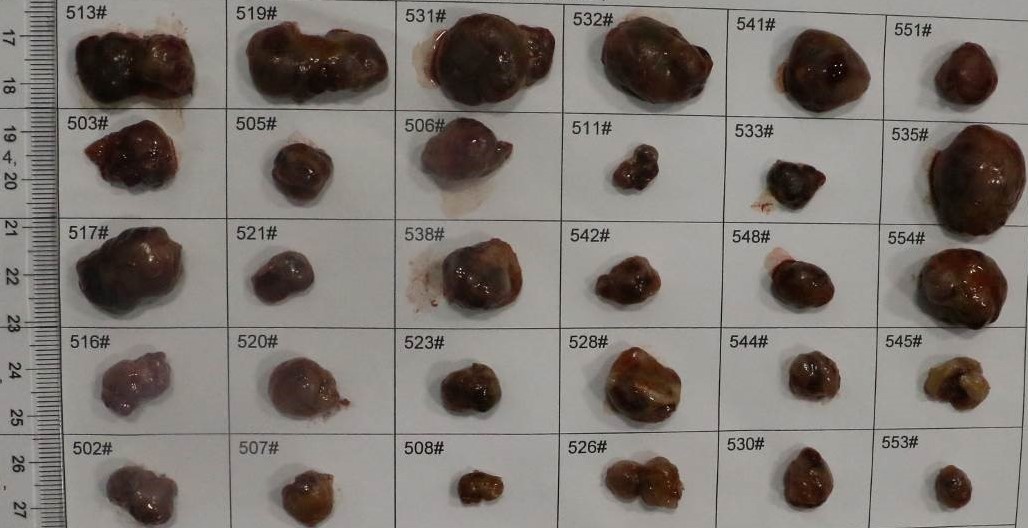** |
| --- | --- |
| Anti-PD-1 Ab  10 mg/kg |  |
| BD0801  0.8 mg/kg |  |
| BD0801  2.5 mg/kg |  |
| BD0801  7.5 mg/kg |  |

Tumor images for Figure 3C

| Vehicle | **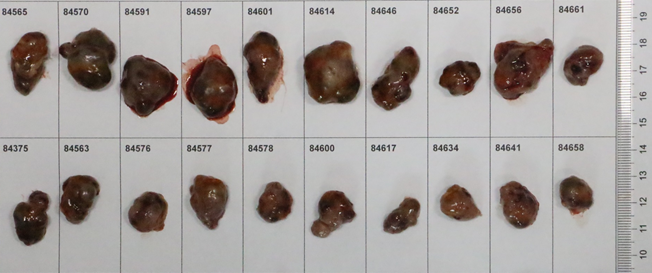** |
| --- | --- |
| BD0801  0.8 mg/kg |  |
| Anti-PD-1 Ab  5 mg/kg | **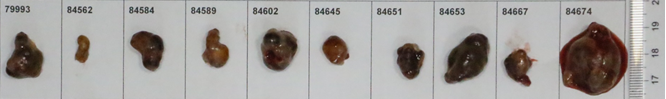** |
| BD0801 0.8 mg/kg + Anti-PD-1 Ab  5 mg/kg | **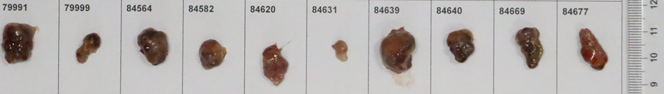** |

Tumor images for Figure 3D

| Vehicle | **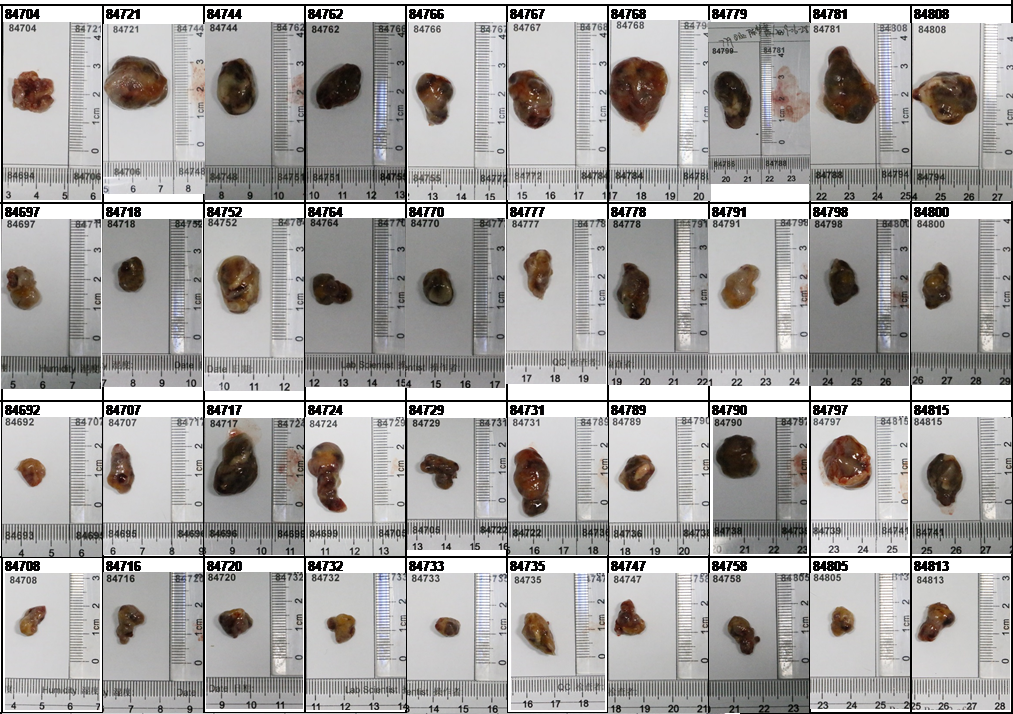** |
| --- | --- |
| BD0801  2.5 mg/kg |  |
| Anti-PD-L1 Ab  5 mg/kg |  |
| BD0801 2.5 mg/kg + Anti-PD-L1 Ab 5 mg/kg |  |

Tumor images for Figure 5

| G1: Vehicle | **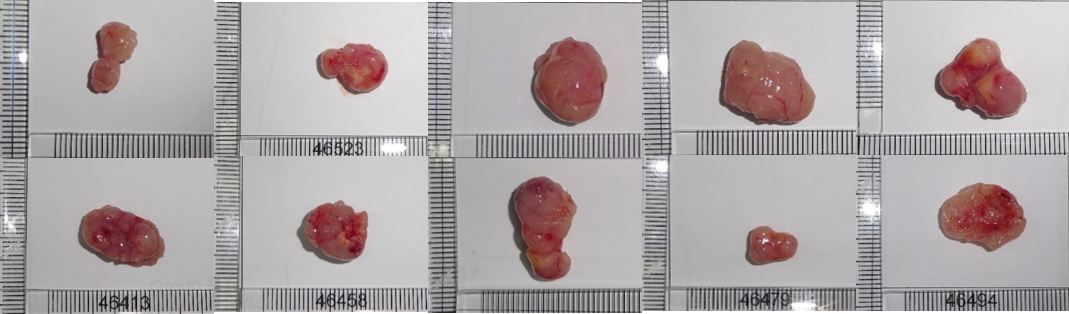** |
| --- | --- |
| G2:  BD0801  0.8 mg/kg | **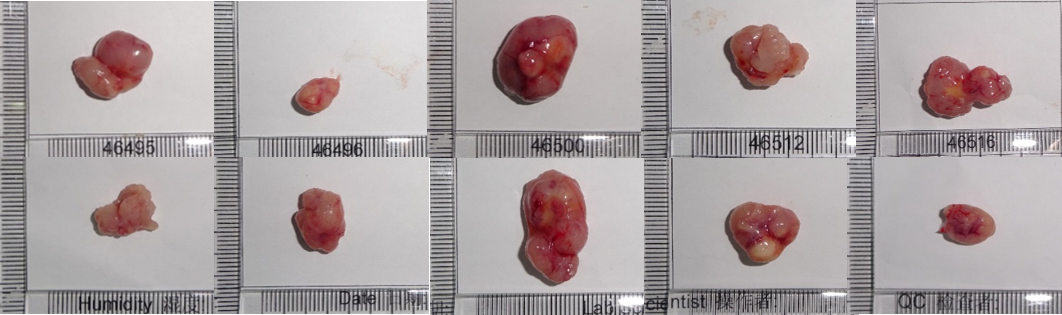** |
| G3:  BD0801  2.5 mg/kg | **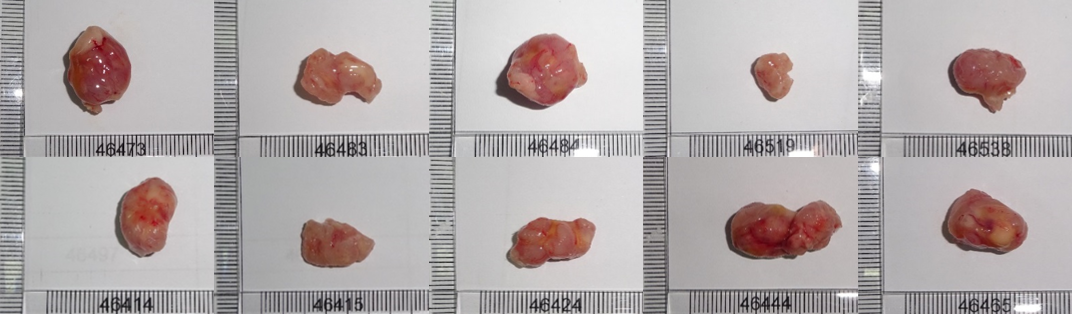** |
| G4:  BD0801  7.5 mg/kg | **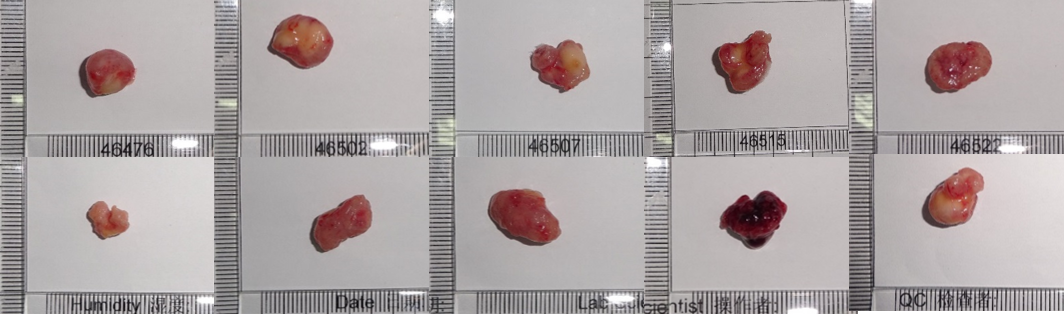** |
| G5:  Anti-PD-1 Ab  10 mg/kg | **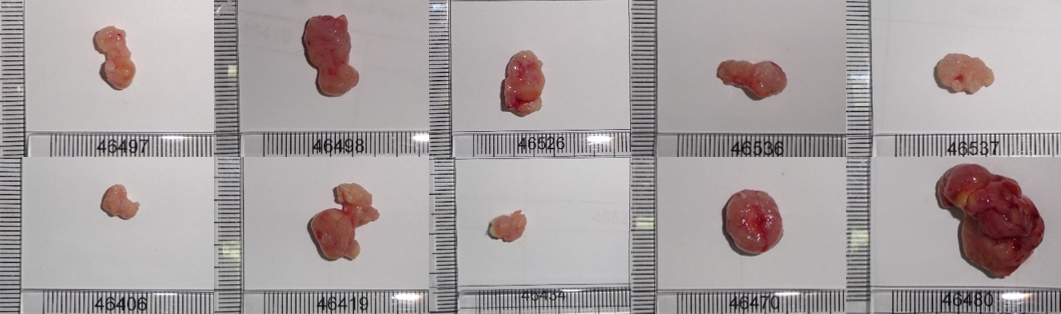** |
| G6:  Anti-PD-L1 Ab  5 mg/kg | **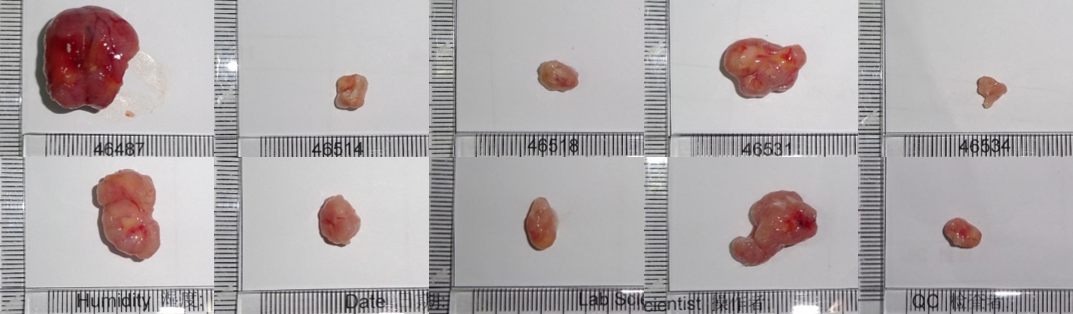** |
| G7:  BD0801  2.5 mg/kg + anti-PD-1 Ab  10 mg/kg | **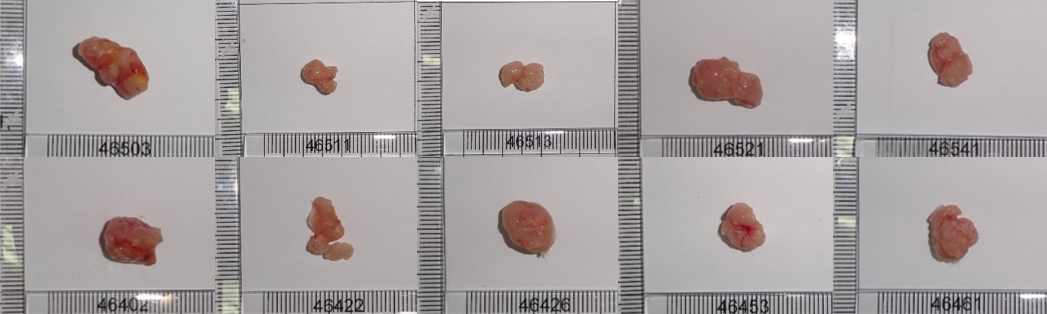** |
| G8:  BD0801  2.5 mg/kg + anti-PD-L1 Ab,  5 mg/kg | **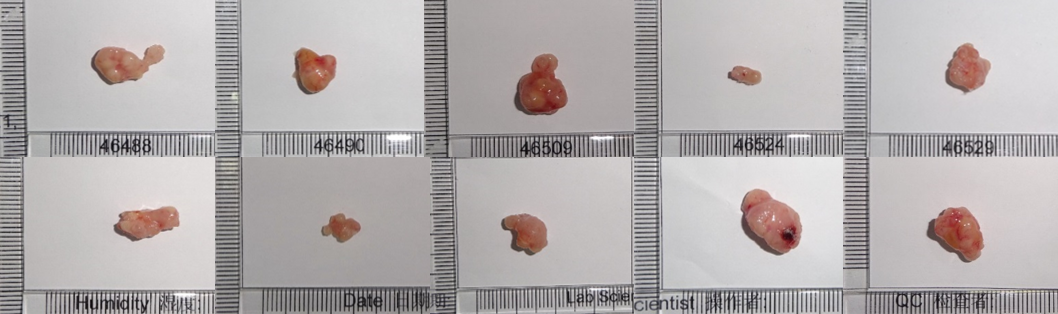** |

**Supplementary Figure S5. Tumor images for** **Figure 2C, 3A, 3C, 3D, and 5.**

**Supplementary Figure S6**

**A**


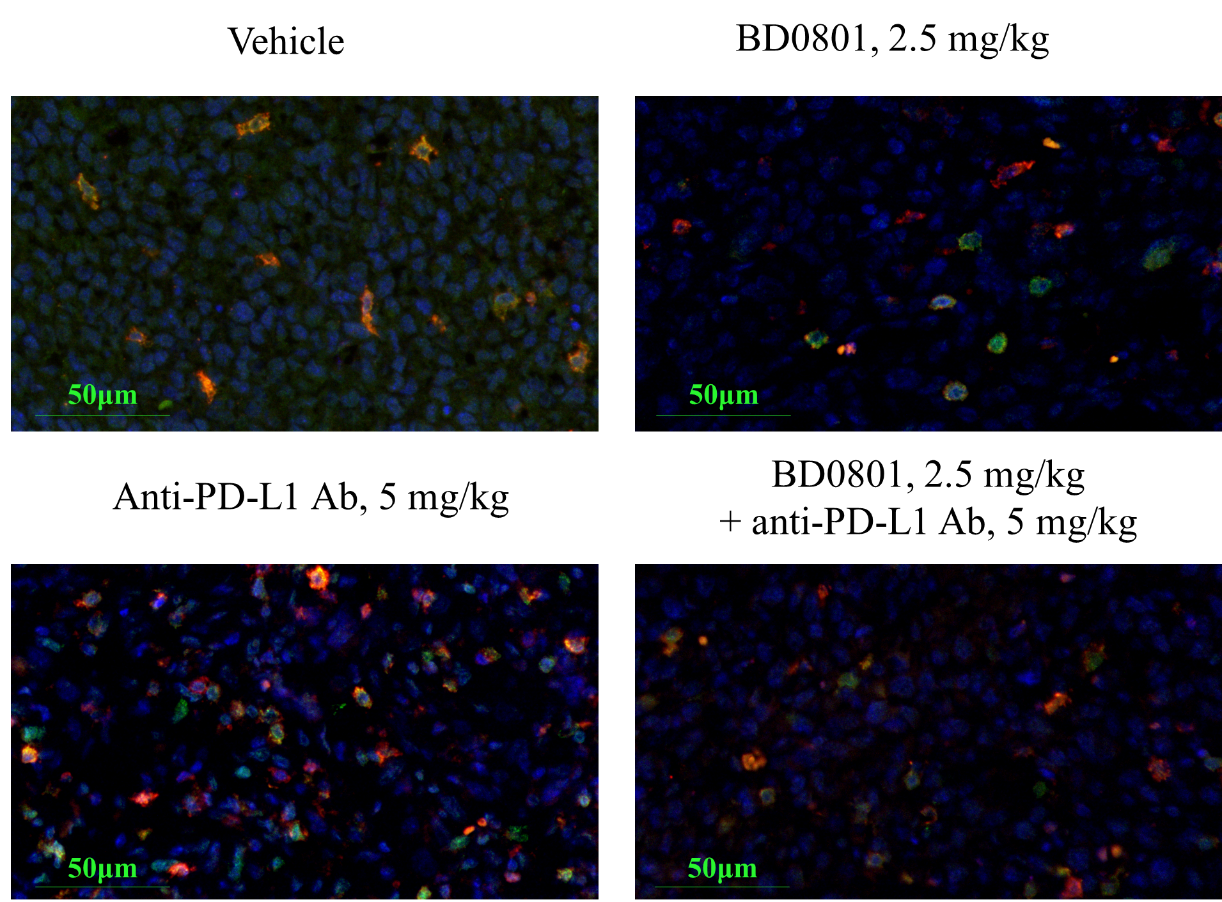


**B**


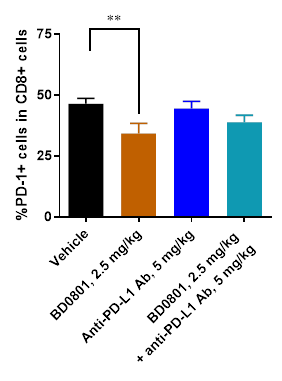


**C**


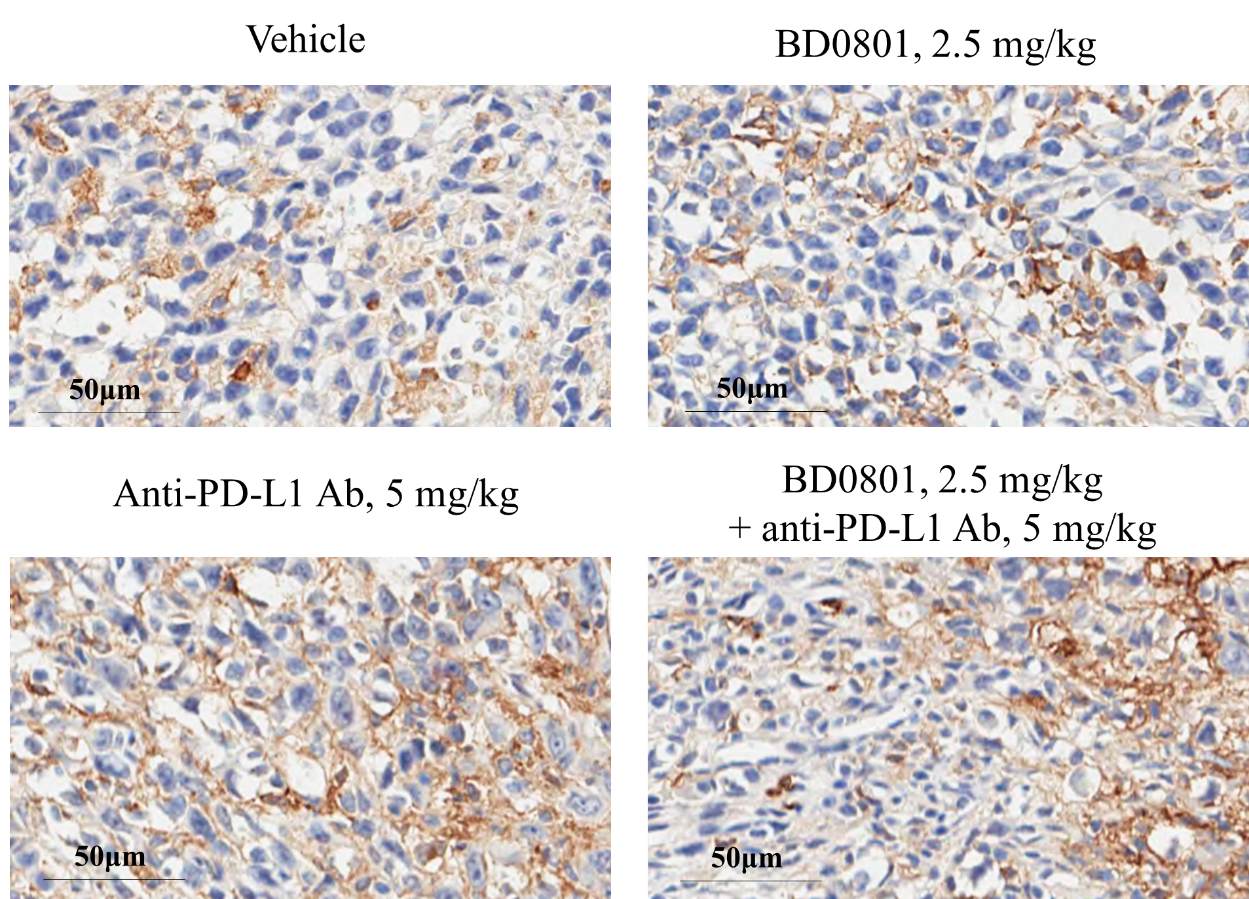


**Supplementary Figure S6. Ex vivo analysis of tumor samples from the 3LL syngeneic mouse model using IF and IHC staining.** (A) The representative images of the IF staining using anti-PD-1 and CD8 antibodies are shown: Red: CD8; Green: PD-1; Blue: DAPI for nuclei (scale bar: 50 μm). (B) The average percentages of the double-positive staining of PD-1^+^CD8^+^ T cells were analyzed (n=9-10/group). (C) The representative images of the IHC staining using anti-PD-L1 antibody are shown (scale bar: 50 μm). The error bars represent SEM; **, P<0.01.

**Supplementary Figure S7**

**A**


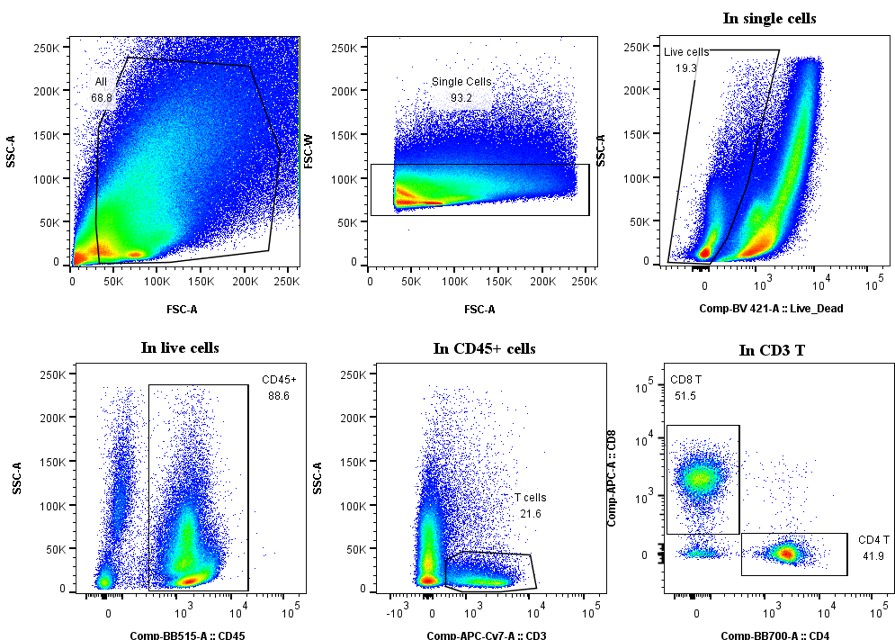


**B**


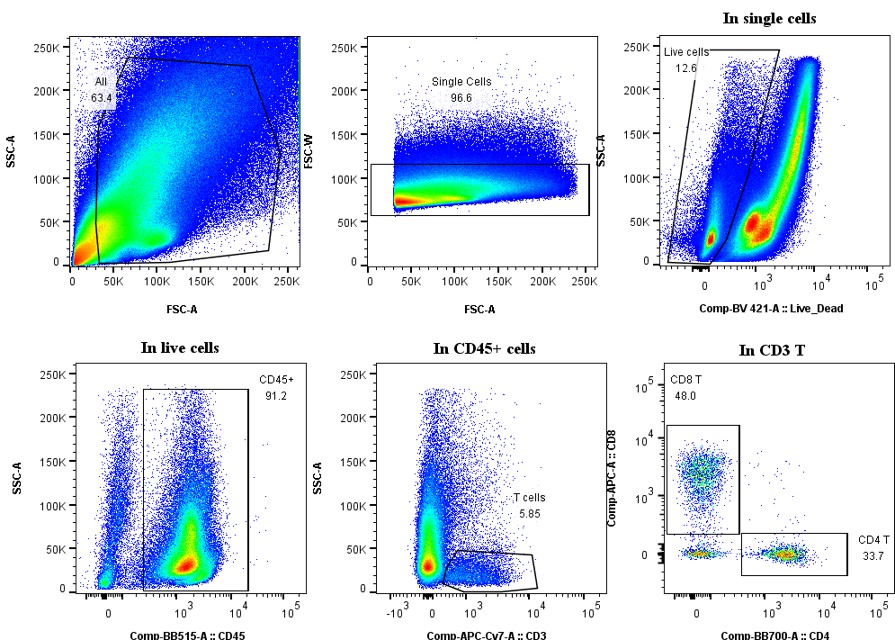


**C**


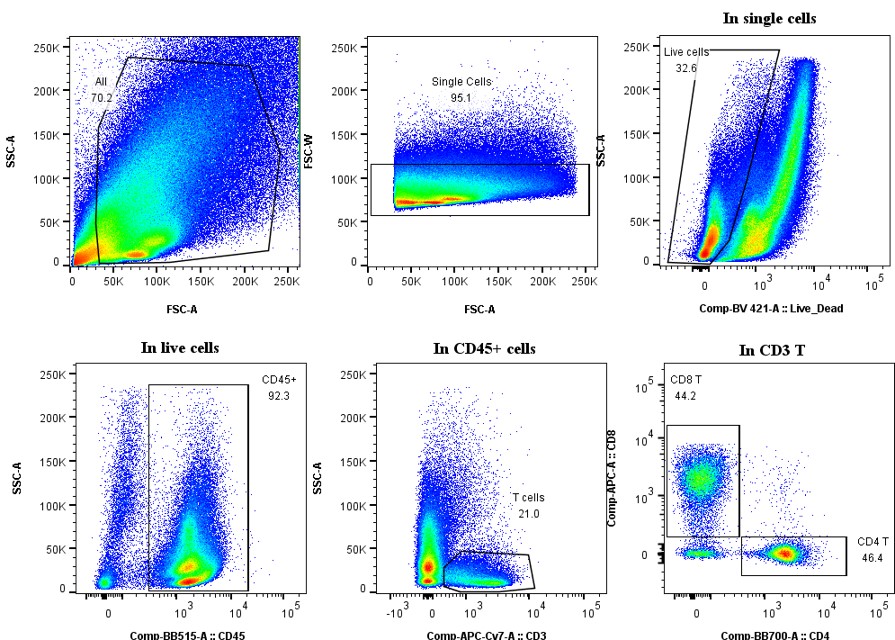


**D**


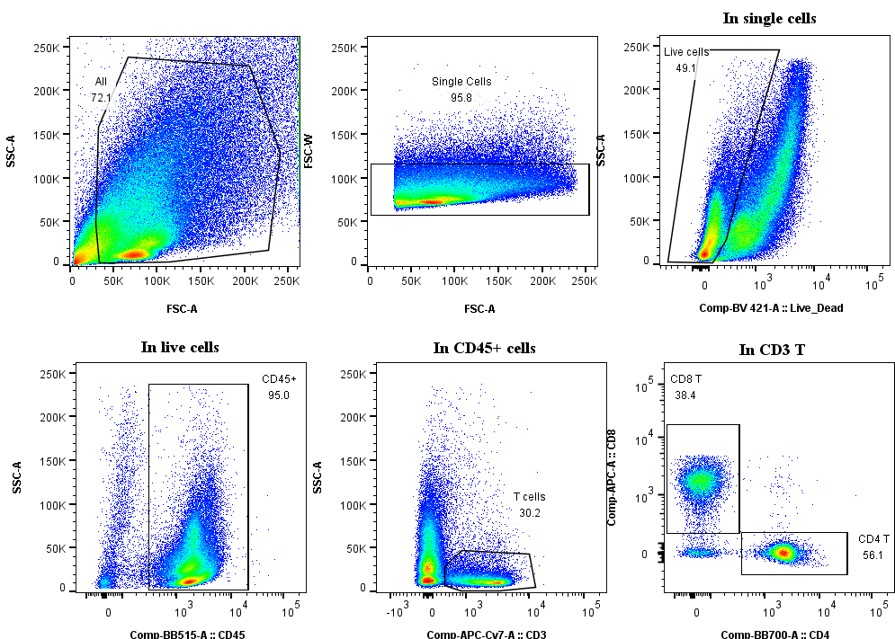


**Supplementary Figure S7. The representative flow images and gating strategy of 4 different groups.** (A) Vehicle. (B) BD0801 2.5 mg/kg. (C) anti-PD-L1 Ab 5 mg/kg. (D) BD0801+anti-PD-L1 Ab.
